# BATF2 inhibits the stem cell-like properties and chemoresistance of gastric cancer cells through PTEN/AKT/β-catenin pathway

**DOI:** 10.7150/thno.98389

**Published:** 2024-10-21

**Authors:** Longlong Cao, Kai Weng, Lujie Li, Guangtan Lin, Yuxuan Zhao, Youxin Gao, Xiaobo Huang, Qiyue Chen, Jiabin Wang, Chaohui Zheng, Changming Huang, Jianwei Xie, Ping Li

**Affiliations:** 1 Department of Gastric Surgery, Fujian Medical University Union Hospital, Fuzhou, Fujian 350001, P. R. China.; 2 Key Laboratory of Gastrointestinal Cancer (Fujian Medical University), Ministry of Education, Fuzhou, Fujian 350001, P. R. China.; 3 Fujian Key Laboratory of Tumor Microbiology, Fujian Medical University, Fuzhou, Fujian 350001, P. R. China.

## Abstract

**Background:** Gastric cancer (GC) ranks as the fifth leading cause of cancer mortality, with cancer stem cells (CSCs) playing a critical role in tumor progression and resistance to chemotherapy. Conventional chemotherapy often fails to effectively target these stem cells. BATF2, a tumor suppressor, is known for its role in gastric cancer, but its influence on cancer stem cell-like properties and chemotherapy response remains unclear.

**Methods:** Single-cell RNA sequencing (scRNA-seq) analysis was performed on 9 gastric cancer samples to evaluate the expression and regulatory function of BATF2. *In vitro* experiments involving cell cultures, tumor cell spheroids, and organoids were conducted to assess BATF2's impact on 5-Fu sensitivity and its interaction with drug transporters and signaling pathways. *In vivo* studies, including subcutaneous tumor formation assays, immunohistochemistry, and immunoblotting, were used to validate findings.

**Results:** BATF2 was confirmed as a tumor suppressor in gastric cancer through scRNA-seq analysis. Elevated BATF2 expression correlated with improved outcomes from postoperative chemotherapy in GC patients and increased sensitivity to 5-Fu. BATF2 enhanced 5-Fu responsiveness by inhibiting the ABCG2 drug transporter and promoting PTEN stability, which suppressed AKT phosphorylation. This led to reduced nuclear β-catenin levels and decreased expression of stem cell markers CD44, SOX2, and NANOG, ultimately reducing chemoresistance and stem-like properties in GC cells.

**Conclusions:** BATF2 plays a pivotal role in regulating stem-like characteristics and chemoresistance in gastric cancer through the BATF2/PTEN/AKT/ABCG2 pathway. These findings suggest a novel therapeutic strategy targeting BATF2 to enhance chemotherapy effectiveness in gastric cancer treatment.

## Introduction

Gastric cancer ranks as one of the top malignant tumors globally, with new cases reaching approximately 0.968 million annually and being the fifth leading cause of cancer-related deaths 1. Notably, about half of these patients receive their diagnosis at an advanced stage where existing treatments fall short in efficacy, leading to dismal survival rates. This situation underscores the critical necessity to deepen our understanding of gastric cancer's origins, pinpoint innovative diagnostic and therapeutic avenues, and foster advancements in precision medicine. Recent scientific inquiries have homed in on cancer stem cells (CSCs), a vital yet minor group of tumor cells distinguished by their ability to differentiate and self-renew 2, 3. These cells typically remain inactive until stimulated by external factors and possess enhanced abilities to resist apoptosis and repair DNA, which allows them to circumvent the effects of standard chemoradiotherapy treatments. Their capacity to continue proliferating even after the primary tumor cells have been eliminated significantly pose significant hurdles in treatment and is a key factor in cancer recurrence 4. The concept of CSCs has been validated in various cancers, like colorectal and breast cancer 5, 6, with common traits including the heightened expression of CD44 7. Therefore, investigating the molecular processes that govern the self-renewal and differentiation of CSCs holds immense theoretical and practical value in advancing cancer treatment.

BATF2, also known as the suppressor of AP-1 or SARI, which is induced by interferon, is located on chromosome 11 in humans. First identified through differential hybridization in 2008, BATF2 is part of the basic leucine zipper (bZIP) protein family 8-10. It features a bZIP domain that inhibits the transcription factor AP-1, influencing various biological processes such as immune responses, growth, development, and inflammation 11, 12. As a tumor suppressor gene, BATF2 plays a pivotal role in the prevention of tumor development and progression, although the specifics of its mechanism are yet to be fully elucidated. In breast cancer research, it has been shown that targeting BATF2 suppresses CCN1 transcriptional activity, thereby reducing tumor cell proliferation and invasion 13. Moreover, BATF2's interaction with plasma ceruloplasmin through its carboxy-terminal domain suggests a mechanism where BATF2 stabilizes binding proteins and modulates the HIF-1α/VEGF pathway, hinting at its broader impact on tumor biology 14. Notably, Xie *et al.* highlighted BATF2's significance in gastric cancer as a suppressor gene, linking its expression to patient prognosis and its potential as a marker for predicting peritoneal metastasis 15. Despite these insights, the relationship between BATF2 and the stem-like traits and chemotherapy resistance in gastric cancer remains underexplored. This study expands on BATF2's role in gastric cancer, demonstrating how it interacts with PTEN to suppress the AKT/β-catenin/ABCG2 signaling pathway, impacting stem cell markers like ABCG2, CD44, SOX2, and NANOG, based on *in vivo* and *in vitro* analyses. These findings underline the potential of targeting gastric cancer stem cell traits via BATF2 to combat chemotherapy resistance and pave new paths for treating gastric cancer.

## Materials and methods

### Human tissue

Between 2010 and 2015, a total of 345 paraffin-embedded samples, including gastric cancer and adjacent non-tumor tissues, were collected at Fujian Medical University Union Hospital (FJMUUH). All tissues were snap-frozen in liquid nitrogen within 30 minutes after surgery and stored long-term. These samples were used for immunohistochemistry analysis. The inclusion criteria were as follows: (1) histological confirmation of gastric cancer, (2) absence of concurrent primary malignancies and distant metastases, (3) completeness of follow-up data, and (4) initial or updated tumor stage classification according to the 7th edition of The American Joint Committee on Cancer Staging Manual. Recurrence was defined as the presence of a biopsy-documented tumor or imaging features and categorized based on location. The present study was approved by the ethics committee of Fujian Medical University Union Hospital (No. 2020KY042), and written consent was obtained from all enrolled patients.

### Tissue microarray (TMA)

A collection of gastric cancer samples embedded in tissue microarray (TMA) was created. The gastric cancer tissues were carefully examined by pathologists, and areas devoid of necrotic and hemorrhagic materials were identified and marked on paraffin blocks. From each sample, a 1.5-mm core was extracted from the donor blocks and inserted into specific array positions in a recipient paraffin block using a TMA instrument. Multiple sequential sections, measuring 4 µm in thickness, were cut from all TMAs. One of these sections was stained with hematoxylin and eosin to serve as a reference.

### RNA-sequencing analysis

Total RNA was extracted from cells using Trizol, chloroform, isopropanol, and absolute ethanol following the instructions of the Trizol kit and RNA extraction manual. The RNA concentration was measured, and cDNA was synthesized using the reverse transcription kit from Mei5 Biotechnology Co., Ltd. Fluorescent quantitative PCR was performed using the qPCR reaction kit (M5 HiPER SYBR Premix EsTaq). The primers used for qPCR were obtained from PrimerBank (https://pga.mgh.harvard.edu/primerbank/). The qPCR reaction conditions were as follows: 95°C for 2 minutes; 40 cycles of 95°C for 30 seconds, 55°C for 30 seconds, and 72°C for 1 minute; followed by 72°C for 10 minutes. Relative quantitative analysis was performed using the 2^-ΔΔCt method (Livak method) to obtain 2^-ΔΔCt, which is directly proportional to the expression level of the target gene.

### Establishment of cell lines

Use lentiviral vectors for overexpression and silencing of BATF2 (NM_138456) purchased from GeneChem Company (Shanghai, China), as well as control lentiviral vectors for experiments. After transfection, culture the cells with penicillin (2µg/mL, Sigma) for 1 week to select successfully transfected cells.

### Western blot analysis

Cells were lysed on ice for 30 minutes in RIPA buffer (Meilunbio, China) containing phenylmethylsulfonyl fluoride (0.1mg/mL). After cell disruption using an ultrasonic cell disruptor and centrifugation, the supernatant was collected. Protein quantification was performed using the Bicinchoninic acid (BCA) analysis kit (Meilunbio, China) with 10µL of the supernatant. Protein samples (40µg) were loaded onto 8%-15% SDS-PAGE gels for electrophoresis. The proteins were then transferred to a Polyvinylidene fluoride (PVDF) membrane (Millipore, USA, ThermoFisher, USA). The PVDF membrane was blocked with a blocking solution (Meilunbio, China) containing 5% skim milk (BD, USA) at room temperature for 1 hour and washed with PBST. The PVDF membrane was incubated with primary antibodies overnight at 4°C, followed by incubation with mouse or rabbit secondary antibodies at room temperature for 1 hour. After washing three times with TBST buffer (Meilunbio, China), the PVDF membrane was detected using Beyo ECL Plus (Beyotime, China, Meilunbio, China) to quantify the grayscale values of bands. The antibodies used in this study are listed in Table S1.

### Tumor spheroid culture

Control or BATF2 stable overexpressing cells from AGS or HGC-27 were cultured in serum-free DMEM/F12 medium in ultra-low attachment 6-well dishes (Corning, USA), supplemented with 20 ng/mL epidermal growth factor, 10 ng/mL basic fibroblast growth factor, 2% B-27 (Life Technologies, Carlsbad, USA), and 2 mM L-glutamine (Life Technologies, Carlsbad, USA). The cultures were maintained at 37℃ in a 5% CO2 incubator for 7 days, with medium changed every 3 days. The diameter and number of tumor spheres were calculated in three random 100x magnification fields using a fluorescence microscope (BZ-X700, Keyence Corp, Atlanta, GA, USA) under bright field model. For the drug experiments, spheroids were treated with 5-Fu at a concentration of 20 µm for 48 hours.

### Human gastric cancer organoid culture

Tumor tissue obtained from the gastric antrum was washed on the epithelial surface with PBS and immediately rinsed in DMEM (11995065, Gibco). The specimen was cut into 2-3mm fragments, and the gastric antrum specimen was placed immediately in a 15mL conical tube containing 10mL preheated gland digestion medium and 1mg/mL collagenase A (C9891, Sigma-Aldrich). The gastric antrum was gently inverted and incubated at 37°C for 20 minutes, followed by centrifugation at 100g for 5 minutes. The supernatant was removed, and the pellet was resuspended in 10mL preheated gland wash medium. The suspension was centrifuged at 100g for 5 minutes, the supernatant was removed, and the pellet was resuspended in 2mL preheated trypsin-EDTA (T3924, Sigma-Aldrich) and then incubated in a 37°C water bath for 4 minutes, with gentle mixing using a 1mL pipette. Trypsin was quenched with 2mL PBS + 0.2% FBS. The suspension was sequentially passed through two 70µm cell strainers, and the flow-through was collected, centrifuged at 1200 RPM for 5 minutes, the supernatant was removed, and the pellet was resuspended in 100-200µL PBS for cell counting. Finally, 50µL of Matrigel™ suspension was carefully added to the center of each well of a 24-well plate, followed by the addition of 0.5mL IntestiCult Organoid Growth Medium (STEMCELL Technologies, Cambridge, USA). The medium was changed twice a week. Starting from the second generation, control or overexpressed BATF2 plasmids were used for lentiviral infection to observe diameter and quantity under a light microscope, randomly selecting three fields of view. After fixation with 4% paraformaldehyde for 1 hour, samples were embedded in 2% agarose gel or directly fixed in formalin in Matrigel to generate paraffin blocks for subsequent slicing and immunofluorescence detection. For the drug experiments, organoids were treated with 5-Fu at a concentration of 20 µm for 48 hours.

### Downloading and analyzing public data resources

The datasets (GSE62254, GSE54129, GSE26253, and GSE15459) were downloaded from the Gene Expression Omnibus (GEO, https://www.ncbi.nlm.nih.gov/gds/). The “Affy” package in R language was used to perform background adjustment on the raw data of the Affymetrix datasets using the robust multichip analysis (RMA) algorithm. The “ComBat” algorithm was used to perform batch effect correction on the raw data of the Illumina datasets.

### Gene Set Enrichment Analysis (GSEA)

To analyze signaling pathways associated with different immune phenotypes, we utilized multiple functional gene sets (Fgs) collected and curated from the Molecular Signatures Database (MSigDB, https://www.gsea-msigdb.org/). The "limma" package in R software was used to analyze differentially expressed genes between immune phenotype tumors and other phenotype tumors. The “clusterProfiler” (v3.12.0) package was employed to calculate the normalized enrichment score (NES) and false discovery rate (FDR) for each gene set. A gene set was considered significant when the FDR was less than 25%.

### Immunohistochemistry

After the tissue chip is baked at 60°C for 60 minutes, xylene is used for dewaxing and gradient ethanol for dehydration. Antigen retrieval is performed using EDTA buffer, and endogenous peroxidase activity is blocked with peroxide. The tissue is fixed with 4% paraformaldehyde and permeabilized with 0.5% Triton X-100 (Sigma, USA). After incubating at room temperature for 60 minutes with serum for blocking, the tissue is separately incubated overnight at 4°C with monoclonal primary antibodies. After washing with PBS buffer, Cy3-conjugated anti-rabbit IgG (Abcam) or FITC-conjugated anti-mouse IgG (Abcam) is applied as the secondary antibody. The samples are incubated at room temperature for 20 minutes, washed with PBS, stained with DAB and counterstained with hematoxylin, followed by ammonia water bluing. The tissue is dehydrated with gradient ethanol and cleared with xylene, mounted with neutral resin for microscopy examination, and observed with a fluorescence microscope. The antibodies used in this study are listed in Table S1.

### Immunofluorescence and confocal imaging

Tissue slices were fixed in 4% paraformaldehyde and permeabilized with 0.5% Triton X-100 (Sigma, USA). The sections were blocked with goat serum in a dark box for 1 day. The slices were incubated with the corresponding primary antibody overnight at 4°C. Cy3-conjugated anti-rabbit IgG (Abcam) and FITC-conjugated anti-mouse IgG (Abcam) were applied as secondary antibodies. Following this, these cells were incubated for 2 hours with the nuclei stained using DAPI (Thermo, USA). Finally, the samples were cover slipped through a fluorescence microscope. Antibodies used in this study are listed in Table S1.

### Tumor formation and metastasis assays

In this study, 4-5-week-old male BALB/c nude mice purchased from Beijing Vital River Laboratory Animal Technology Co., Ltd. were used. Each nude mouse was injected with 5 × 106 stably transfected cells into the right axillary fossa. After 7 days of inoculation, all mice were divided into control and treatment groups (five mice per group). The control group received DMSO dissolved in corn juice (as a control), while the treatment group received 5-Fu (MCE, 2 mg/kg/3 days). Tumor volume was measured every 3 days and calculated using the formula V = (L × W2)/2 (V, tumor volume; L, length; W, width). After 3-4 weeks of injection, the mice were euthanized, and the tumors were weighed. All animal experiments were conducted in accordance with the regulations of the Animal Protection Committee of Fujian Medical University and approved by the Ethics Committee of Fujian Medical University/Laboratory Animal Center.

### Flow cytometry

The novel tumor tissue is cut into 2-4mm fragments and added to a mixture of type I collagenase diluted with PBS. The mixture is then placed in a 37°C, 360°C shaking incubator for 40 minutes. The cell suspension is centrifuged at 400g for 5 minutes, the supernatant is removed, and the cells are resuspended in PBS. The cells are then gently dispersed using a pipette and filtered to remove cell clumps and fragments. The cell suspension is collected in a centrifuge tube, centrifuged at 400g for 5 minutes, the supernatant is removed, and the cells are resuspended and centrifuged again. The cells are then resuspended in 500µl of flow cytometry staining buffer for counting and viability analysis. Different fluorescently labeled antibodies are sequentially added to the cell suspension based on cell number. The mixture is incubated at room temperature for 30 minutes (with a blank control group set up at the same time). The suspension is then centrifuged at 1200rpm for 5 minutes, the supernatant is discarded, and 1ml of PBS buffer is added to wash the cells twice. Finally, the cells are resuspended in 500µl of PBS in a flow cytometry tube, covered with foil to avoid light exposure, and analyzed on a flow cytometer.

### Statistical analysis

The statistical analysis of quantitative data sets was performed using GraphPad Prism software. The data are presented as means ± standard error (SE) and were analyzed using Student's t-test and one- or two-way analysis of variance (ANOVA) with Dunnett's post hoc analysis. Statistical significance was set at P<0.05. In all panels, *p< 0.05, **p < 0.01 and ***p < 0.001.

### Data availability statement

The datasets used and/or analyzed in this study are available upon request from the corresponding author.

## Results

### Decreased BATF2 expression correlates with poor survival and stem cell-like properties in GC

**Figure 1:**
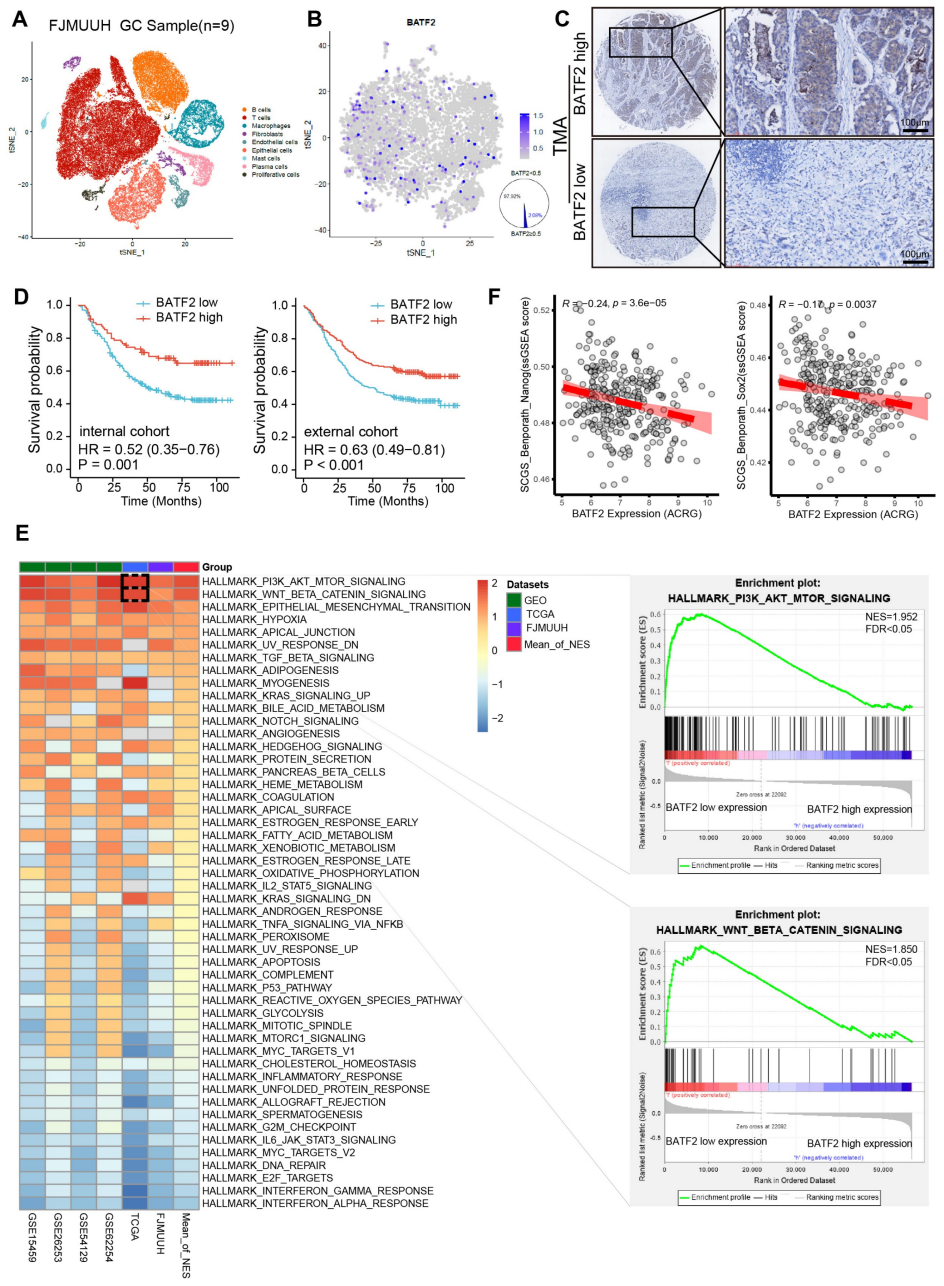
** Decreased BATF2 expression correlates with poor survival and stem cell-like properties in GC.** (A) t-SNE visualization of cell types across 56,693 cells from nine gastric cancer tissue samples. (B) FeaturePlot distinguishing BATF2-positive (blue) from BATF2-negative (gray) epithelial cells. (C) Images showcasing various levels of BATF2 immunohistochemistry (IHC) staining in gastric cancer samples. (D) Kaplan-Meier survival analysis associating BATF2 expression levels to overall survival in a 345-patient internal cohort a 495-patient external cohort. (E) Heatmap of GSEA comparing low versus high BATF2 expression across TCGA, GEO, and FJMUUH cohorts. (F) Pearson's correlation analyses between BATF2 mRNA level and single-sample gene set enrichment analysis (ssGSEA) scores for two verified stem cell signatures, namely Stem cell gene set (SCGS)_Benporath_Nanog and SCGS_Benporath_Sox2, in ACRG cohort.

To examine the specific expression levels of BATF2 in human gastric cancer specimens, we incorporated nine samples into our single-cell RNA sequencing (scRNA-seq) study (Table S2). After filtering out low-quality cells, a total of 56,693 cells were analyzed. These cells were initially categorized into 9 distinct cell types (Figure 1A, Figure S1A-B). Among epithelial cells, a mere 2.08% were BATF2 positive, in contrast to the 97.92% that were BATF2 negative (Figure 1B). Furthermore, within our center's gastric cancer cohort, there were 250 instances of low BATF2 expression versus 95 cases with high expression (Figure 1C). Survival analysis indicated better outcomes for patients with high BATF2 expression, a trend consistent in both internal and external patient groups (Figure 1D). Additionally, an integrated analysis was performed using whole-transcriptome sequencing data from 48 human gastric tumor tissues in our center, combined with public datasets from The Cancer Genome Atlas (TCGA) and Gene Expression Omnibus (GEO) databases (GSE62254, GSE54129, GSE26253, and GSE15459). Of note, subsequent GSEA Hallmark gene set enrichment analysis revealed robust activation of the PI3K/AKT/MTOR, WNT/β-catenin and Epithelial-Mesenchymal transition pathways in specimens with low BATF2 expression compared to those with high BATF2 expression (Figure 1E). These pathways have been shown to be highly associated with tumor cell stemness 16, 17. Consistently, stem cell differentiation, stem cell population maintenance, and PI3K/AKT/MTOR signaling were significantly enriched in epithelial cells with low BATF2 expression, compared to high expression in scRNA-seq data (Figure S1C). Pearson's correlation analyses revealed that BATF2 expression levels negatively correlated with single sample GSEA (ssGSEA) scores for two verified stem cell signatures, namely Stem Cell Gene Set (SCGS)_ Benporath_Nanog and SCGS_Benporath_Sox2 (Figure 1F). Taken together, these results indicate that the downregulation of BATF2 correlates with poor survival and stem cell-like properties in GC.

### BATF2 inhibits stem cell-like properties in gastric cancer spheroids and organoids

**Figure 2:**
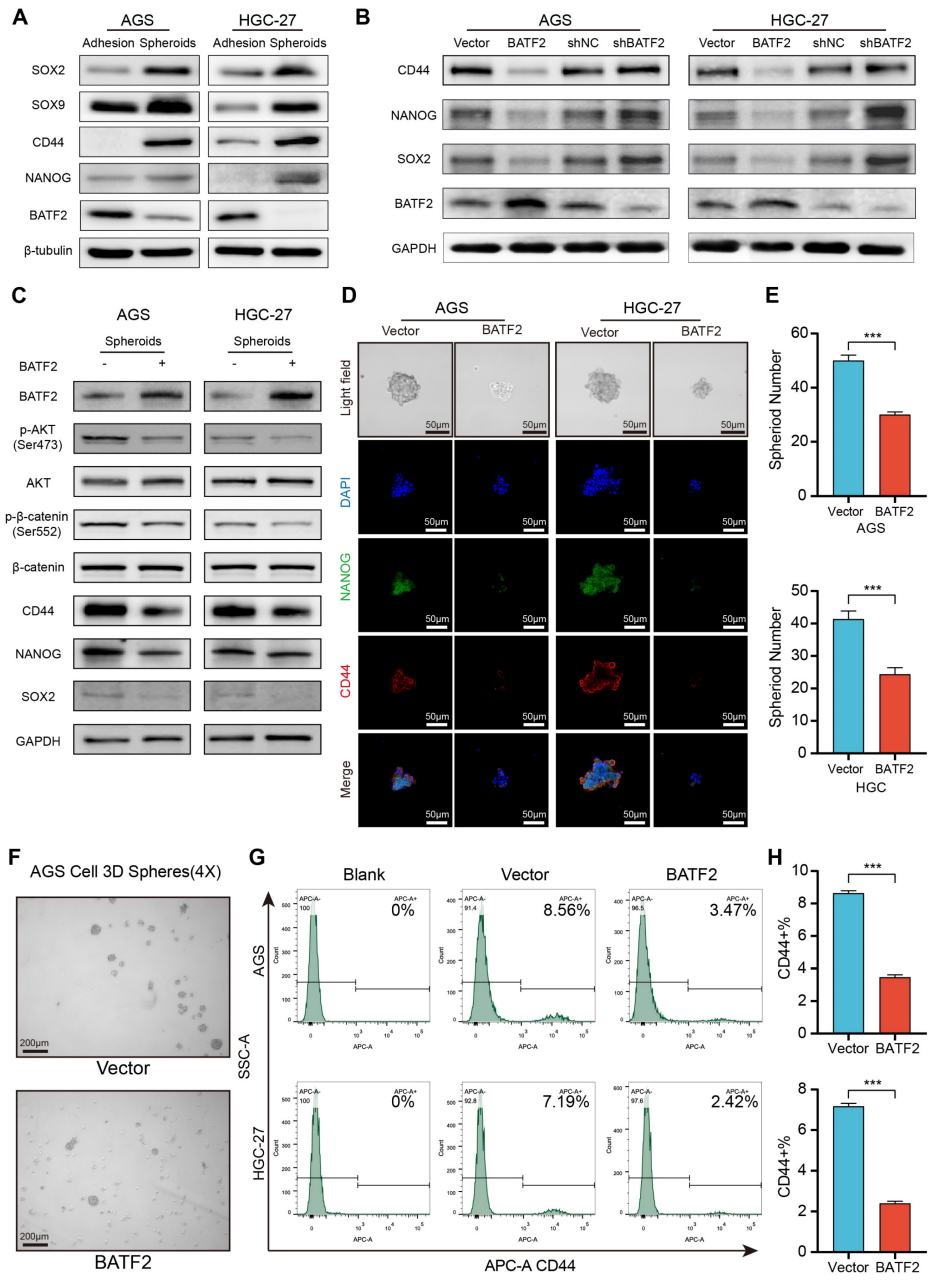
** BATF2 inhibits stem cell-like properties in gastric cancer spheroids.** (A) Analysis of stem cell marker gene expression in adherent and spheroid cells via western blot. (B) Examination of stem cell markers in AGS and HGC-27 cells with BATF2 overexpression or knockdown. (C) Western blot assessment of stem cell markers and AKT pathway components in AGS and HGC-27 spheroid with BATF2 overexpression. (D-E) Immunofluorescence imaging of CD44 and NANOG in spheroids, accompanied by quantifications of spheroid number. (F) Visual comparison of spheroids with and without BATF2 overexpression. (G-H) Flow cytometric evaluation of CD44-positive cells in AGS and HGC-27 cells with and without BATF2 overexpression.

To investigate BATF2's influence on the stem-like characteristics of gastric cancer cells, we assessed the presence of stem cell-associated markers in both traditional adherent cell cultures and 3D tumor spheroids. In the 3D spheroids derived from AGS and HGC-27 cell lines, stemness markers such as CD44, SOX2 and NANOG were markedly elevated compared to their levels in adherent cells at the protein and mRNA levels, while BATF2 levels were reduced (Figure 2A, Figure S2A). When BATF2 was overexpressed in adherent AGS and HGC-27 cells, a noticeable decline in the stem cell markers CD44, NANOG, and SOX2 was observed, whereas reducing BATF2 expression led to an increase in these markers (Figure 2B, Figure S2B). Enhancing BATF2 expression in spheroids from these cell lines inhibited the AKT/β-catenin signaling and decreased CD44 and NANOG levels (Figure 2C-D, Figure S2C), and resulted in fewer spheroids, mirroring the control group's outcomes (Figure 2E-F). Flow cytometry analysis showed a decrease in the population of CD44-positive cells with BATF2 overexpression, consistent with the aforementioned results (Figure 2G-H). These findings collectively demonstrate BATF2's capability to suppress the stem-like characteristics in gastric cancer cells.

We hypothesized that BATF2 inhibits stem-like properties of gastric cancer cells via AKT signaling pathway. To test this hypothesis, we conducted a rescue experiment where BATF2 knockdown-induced overexpression of SOX2, CD44, and NANOG was abolished by MK2206 treatment, an AKT pathway inhibitor (Figure S3A). In these cells, forming 3D tumor spheres, we observed elevated markers of stem cell-like traits and an increase in p-AKT and its target genes in the BATF2-knockdown (shBATF2) group, suggesting a link with AKT signaling (Figure S3B). Compared to controls, shBATF2 cells formed more and larger 3D spheres (Figure S3C). Immunofluorescence on these 3D spheroids (Figure S3D) showed the similar results with the aforementioned western blotting (Figure S3A), indicating BATF2's role in controlling stem cell-like characteristics through AKT signaling. This was supported by statistical analysis (Figure S3E). Flow cytometry further confirmed an increased proportion of CD44-positive cells in the shBATF2 group, aligning with prior observations (Figure S3F-G). Notably, MK2206 mitigated the shBATF2-induced enhancement of stem-like properties, underscoring the role of the AKT pathway in this process (Figure S3D-G). Collectively, these results illustrate that BATF2 knockdown amplifies gastric cancer stem cell-like properties by upregulating the AKT pathway and its associated stemness-related genes.

**Figure 3:**
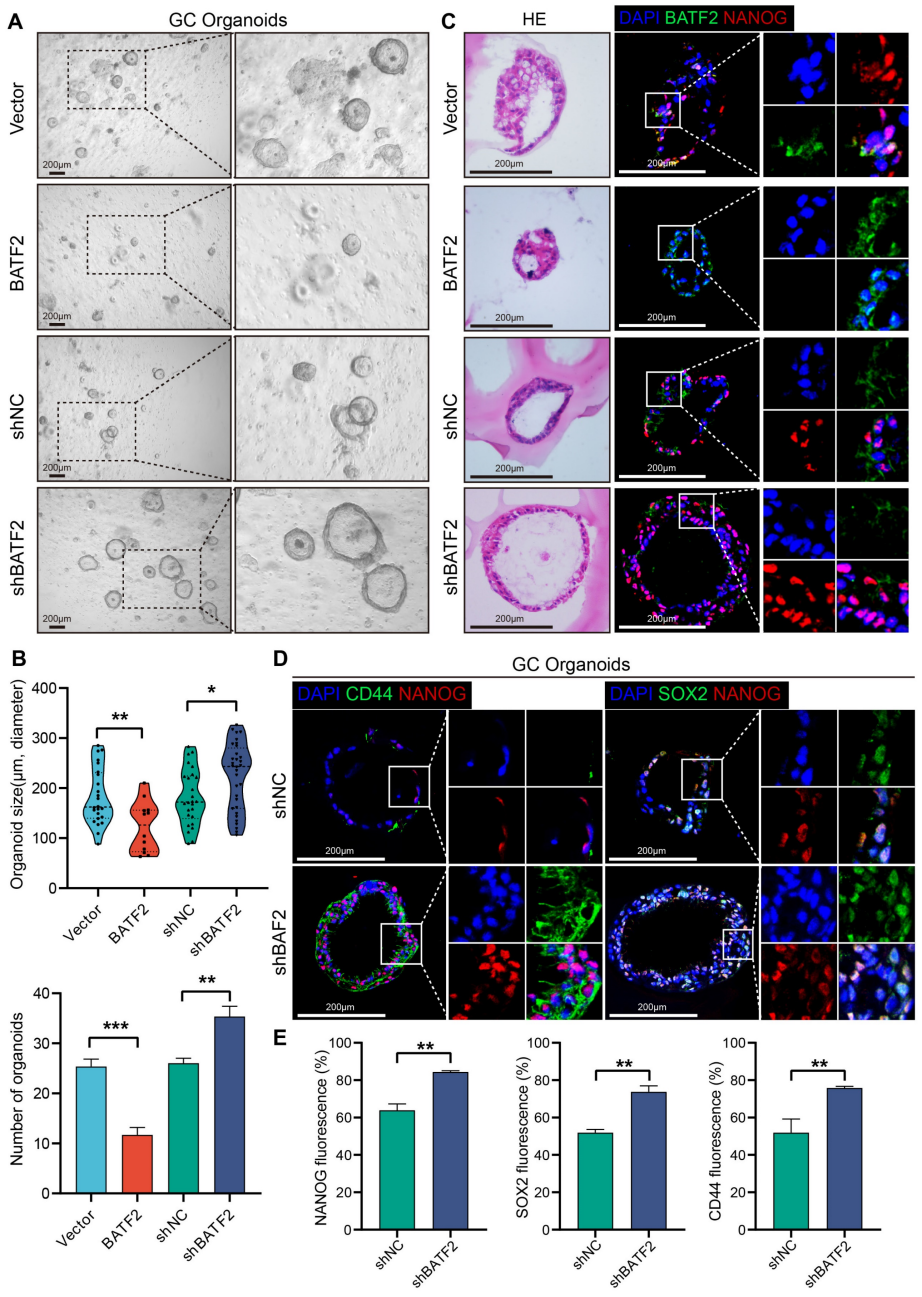
** BATF2 inhibits stem cell-like properties in gastric cancer organoids.** (A-B) Visualization and measurement of organoid numbers and sizes with BATF2 overexpression or knockdown. (C) Immunofluorescence and Hematoxylin&eosin staining for BATF2 and NANOG in organoids following BATF2 modulation. (D-E) Immunofluorescence imaging of CD44 and NANOG in organoids with BATF2-knockdown and their numerical analysis.

In addition to the adherent cell and 3D tumor spheroids, organoids, which closely mimic the tumor microenvironment, serve as an invaluable *in vitro* tool for studying gastric cancer's stem-like characteristics. When gastric cancer organoids were infected with the BATF2-expressing letiviruses, there was a notable decline in both the count and size of these organoids after 14 days, compared to the control group (Figure 3A-B). This reduction underscores BATF2's significant role in controlling the proliferation of gastric cancer organoids. Conversely, organoids infected with shBATF2 exhibited an increase in number and size, suggesting that reducing BATF2 intensifies the stem-like traits of the organoids (Figure 3A-B). Additionally, organoids with BATF2 overexpression showed a marked decrease in nuclear NANOG staining, while those with reduced BATF2 exhibited increased nuclear NANOG levels compared to controls (Figure 3C). The expression of CD44 and SOX2 was also significantly higher in the BATF2 knockdown organoids than in the control group (Figure 3D-E). These observations affirm BATF2's role as a tumor suppressor that mitigates stem cell-like features in gastric cancer organoids by suppressing SOX2, CD44, and NANOG expression.

### Elevated expression of BATF2 hampered gastric tumorigenesis

**Figure 4:**
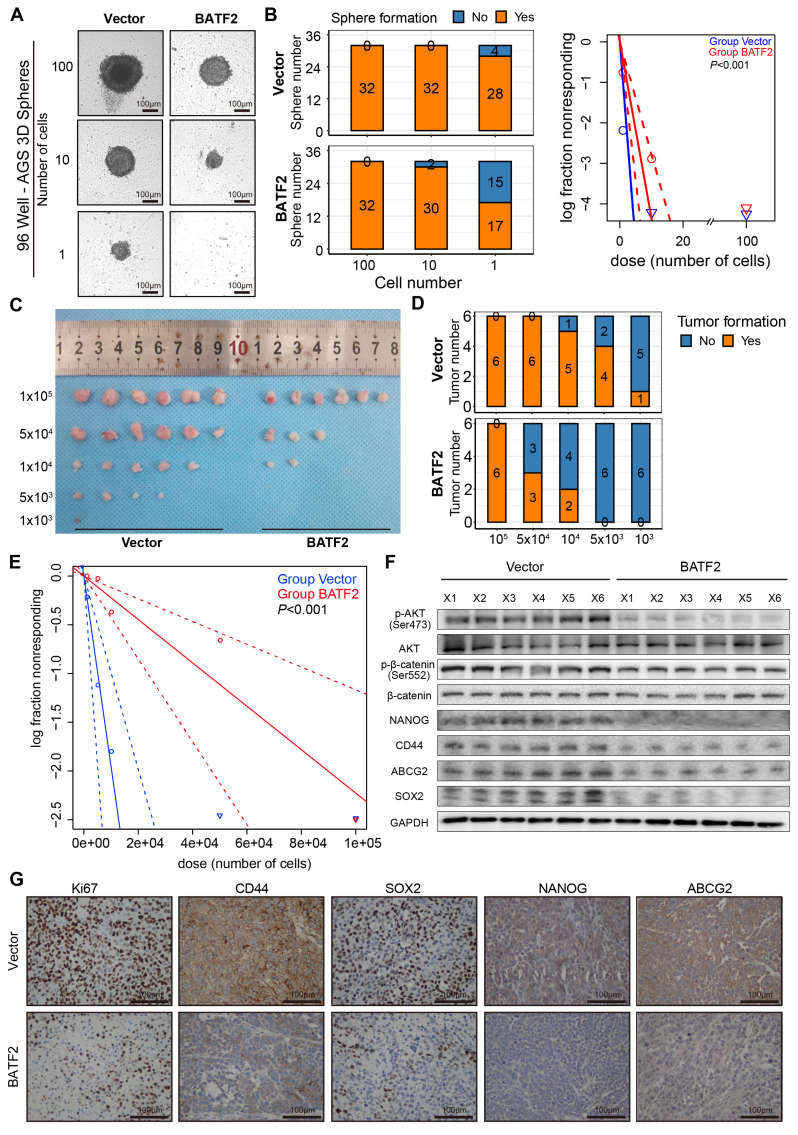
** Elevated expression of BATF2 hampers gastric tumorigenesis.** (A-B) Spheroid formation frequency in U-bottom 96-well plates using AGS cells with varying BATF2 expression. (C-E) Serial dilution and subcutaneous xenografting of AGS cells with/without BATF2 overexpression into NOD/SCID mice, tracking tumor cell injection numbers and tumor formation frequency by day 42, with probability estimates from Extreme Limiting Dilution Analysis (ELDA). (F) Western blot analysis for stem cell markers and AKT pathway elements in xenograft tumors. (G) IHC staining for Ki67, CD44, SOX2, NANOG, and ABCG2 in xenograft tumor samples.

We extended to evaluate BATF2's impact on spheroid and tumor formation, employing dilution assays with AGS and MKN-45, which were confirmed as BATF2 low expression cell line (Figure S4A-C), to assess the influence of BATF2 overexpression both *in vitro* and *in vivo*. The *in vitro* findings demonstrated a notable decrease in spheroid formation among AGS and MKN-45 cells with BATF2 overexpression (Figure 4A-B, Figure S4D-F). To further explore BATF2's role in modulating stem-like characteristics of gastric cancer cells in a live organism, we developed tumor xenograft models using various cell counts of BATF2-overexpressing and control cells. The *in vivo* experiments showed that a lower number of BATF2-overexpressing tumor cells failed to initiate tumor formation compared to control cells (Figure 4C-E, Figure S4G-I), suggesting a diminished stem-like potential in cells with elevated BATF2 levels. Further analyses, including western blot and immunohistochemistry, demonstrated a reduction in AKT/β-catenin signaling activity, aligning with the observed inhibition of tumor growth (Figure 4F-G). These results collectively underscore BATF2's crucial function in curtailing the stem-like proliferation capabilities of cancer cells during the development of gastric tumors.

### BATF2 enhances the sensitivity of 5-Fu chemotherapy in gastric cancer

**Figure 5:**
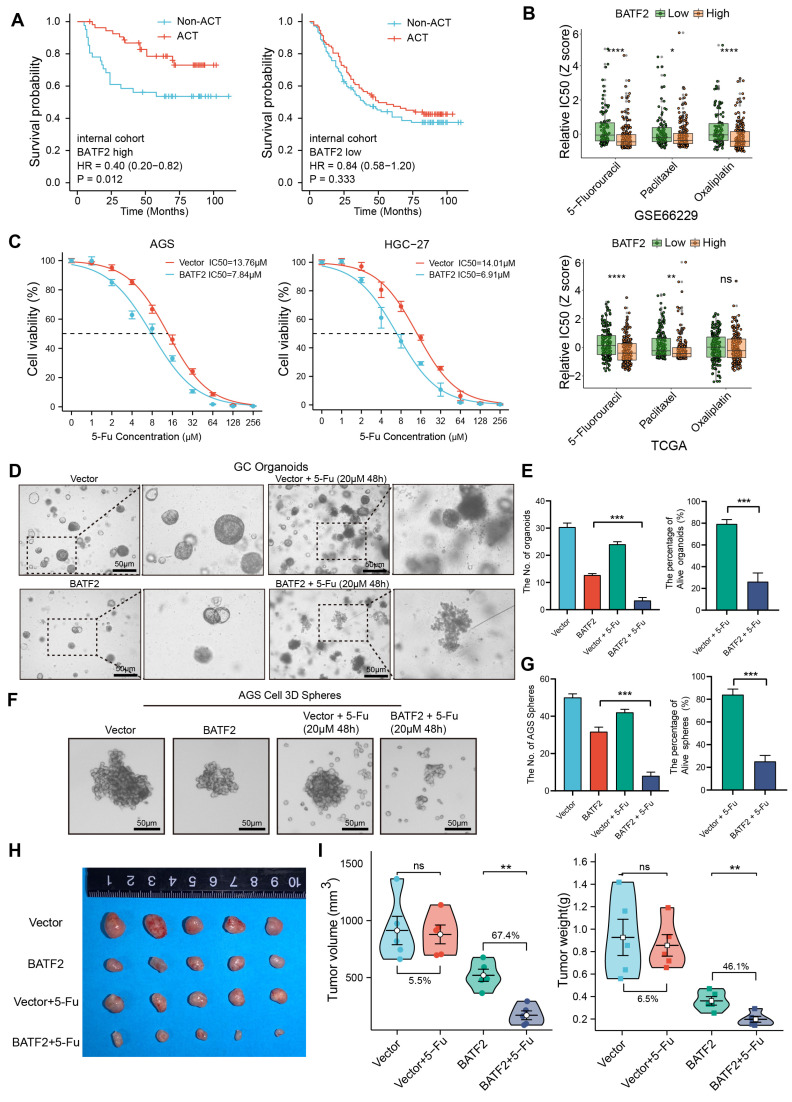
** BATF2 enhances the sensitivity of 5-Fu chemotherapy.** (A) Kaplan-Meier survival analysis assessing chemotherapy's impact based on BATF2 high and low expression patients in internal cohort. (B) Relative half-maximal inhibitory concentration (IC50) was predicted for samples from TCGA and GEO cohorts based on various BATF2 expressions via the oncoPredict package in R software. (C) Concentration-survival curves for 5-Fu in BATF2-overexpressing AGS and HGC-27 cell lines determined by CCK-8 assay. (D-E) Images and analysis of gastric cancer organoids treated with BATF2 overexpression and/or 5-Fu (20μM 48h), showing viable organoid counts. (F-G) Images and quantification of viable AGS 3D tumor spheres post BATF2 overexpression and/or 5-Fu treatment (20μM 48h). (H) Tumor morphology in a NOD/SCID mouse model treated with BATF2-overexpressing AGS cells and/or 5-Fu. Mice received 5-Fu (2 mg/kg/3 days, intraperitoneal injection) (n = 5 tumors per group). (I) Comparative tumor volume and weight post-treatment.

Cancer stem cells are typically accompanied by chemotherapy resistance. Subsequently, we investigate the impact of BATF2 on chemotherapy resistance. High BATF2 expression correlated with improved survival post-adjuvant chemotherapy (ACT). However, in patients with low BATF2 expression, chemotherapy did not improve their prognosis (Figure 5A, Figure S5A). We used the oncoPredict tool to predict the relative half-maximal inhibitory concentrations (IC50) in samples from TCGA and GEO databases, and confirmed high BATF2 expression's link to increased 5-Fu sensitivity (Figure 5B). Our findings align with previous chemotherapy data, suggesting BATF2's role in enhancing gastric cancer cells' responsiveness to 5-Fu (Figure 5C). For instance, in AGS cells, the 5-Fu IC_50_ value dropped from 13.76 µM to 7.84 µM with BATF2 overexpression, and in HGC-27 cells, it decreased from 14.01 µM to 6.91 µM. In addition, we developed 5-Fu resistant (5-Fu/R) AGS and HGC-27 cell lines. Western blotting revealed that the AKT/β-catenin signaling and its downstream stemness markers was activated in the 5-Fu/R AGS and HGC-27 cells compared to the parental cells, whereas the overexpression of BATF2 could reduce their expression (Figure S5B). Cell cytotoxicity experiments showed that in HGC-27 5-Fu/R cells, the 5-Fu IC_50_ value dropped from 62.99 µM to 31.79 µM with BATF2 overexpression, and in AGS 5-Fu/R cells, it decreased from 72.53 µM to 28.58 µM (Figure S5C). Further validation came from studies on organoids (Figure 5D-E) and 3D tumor spheres (Figure 5F-G) derived from gastric cancer tissues and cell lines. These models, when modified to overexpress BATF2, displayed reduced size and number, and showed enhanced sensitivity to 5-Fu therapy compared to their control counterparts. An *in vivo* tumor xenograft model further verified that BATF2 overexpression combined with 5-Fu treatment led to a 67.4% reduction in tumor growth, without affecting body weight, compared to a minimal effect in control cells with overexpression of BATF2 (Figure 5H-I, Figure S5D). These results suggest that BATF2 can significantly enhance the efficacy of 5-Fu therapy in treating gastric cancer.

### BATF2 increases 5-Fu sensitivity via blocking PTEN/AKT/β-catenin/ABCG2 signaling

**Figure 6:**
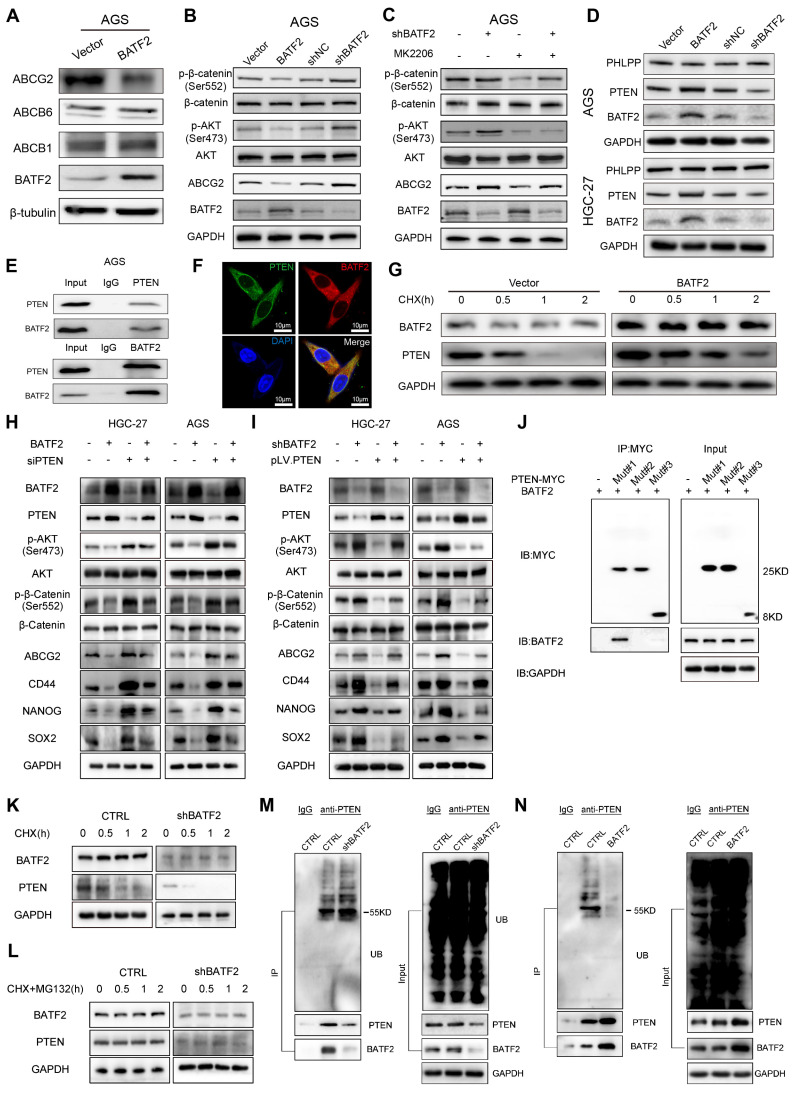
** BATF2 increases 5-Fu sensitivity via blocking PTEN/AKT/β-catenin/ABCG2 signaling.** (A) Western blot analysis of ABCG2, ABCB6, and ABCB1 in AGS cells with elevated BATF2 expression. (B) Western blot analysis of AKT signaling components in BATF2-modified AGS cells. (C) Protein level assessment of AKT pathway elements in AGS cells treated with MK2206 (D) Western blot for PTEN and PHLPP in BATF2-altered AGS and HGC-27 cells. (E) Co-immunoprecipitation assay exploring the interaction of BATF2 and PTEN. (F) Immunofluorescence staining showing PTEN (green) and BATF2 (red) colocalization in gastric cancer cells, with DAPI (blue) nuclear staining. (G) Western blot analysis of PTEN protein stability in AGS cells after cycloheximide treatment. (H) Western blot of stem cell markers and key AKT pathway components in GC cell lines with stable BATF2 overexpression, followed by PTEN deletion using siRNA. (I) Western blot of stem cell markers and key AKT pathway components in BATF2 stable knockdown GC cell lines transfected with pLV.PTEN. (J) Representative IP analysis of the interaction of truncated PTEN proteins and BATF2 in lysates of AGS cells. (K) Effect of BATF2 depletion on PTEN stability in AGS cells incubated with CHX for the indicated times. (L) Effect of MG-132 on PTEN stability regulated by BATF2 knockdown. AGS cells were incubated with 20 μM CHX plus 10 μM MG-132 at the indicated time points. (M) Effect of BATF2 depletion on PTEN ubiquitination in AGS cells incubated with 10 mM MG-132. (N) Effect of BATF2 overexpression on PTEN ubiquitination in AGS cells incubated with 10 mM MG-132.

To explore how BATF2 heightens the responsiveness of gastric cancer cells to 5-Fu therapy, we performed whole-transcriptome sequencing which suggested a link between BATF2 and the ATP-binding cassette transporter, pinpointing ABCG2 as a key downstream target influenced by BATF2 (Figure S6A-C). Known for its role as a multidrug transporter, ABCG2 contributes to the pharmacokinetic properties of drugs and is implicated in the multidrug resistance phenomenon in cancer cell 18. Our subsequent experiments, including western blotting and qPCR, demonstrated that BATF2 specifically suppressed the expression of the ABCG2 protein, without affecting ABCB1 and ABCB6 (Figure 6A, Figure S7A-B). Single-cell sequencing data reinforced this conclusion, showing a clear segregation between BATF2-positive and ABCG2-positive cells in epithelial cells (Figure S7C), highlighting a negative correlation between BATF2 and ABCG2. Intriguingly, as 5-Fu concentrations increased, we observed a rise in ABCG2 expression coupled with a decline in BATF2 levels (Figure S7D) suggesting a dynamic interaction between these proteins. This pattern indicates that gastric cancer cells with high BATF2 levels exhibit increased vulnerability to chemotherapy, while those surviving tend to resist drugs, as indicated by heightened ABCG2 levels. Consistent with prior phosphokinase research, BATF2 expression markedly influenced AKT phosphorylation 15. Western blot analysis further elucidated BATF2's role in modulating AKT and β-catenin phosphorylation, impacting ABCG2 expression, a key protein in drug resistance (Figure 6B, Figure S7E). In AGS and HGC-27 cells with reduced BATF2, the AKT inhibitor MK2206 significantly lowered p-AKT and related protein levels, suggesting BATF2's regulatory effect upstream of AKT (Figure 6C, Figure S7F). Organoid immunofluorescence and protein partitioning studies indicated that BATF2 overexpression decreases β-catenin's nuclear presence, in contrast to BATF2 knockdown effects (Figure S7G-I). BATF2 was observed to increase PTEN expression without affecting PHLPP levels (Figure 6D, Figure S7J). Co-immunoprecipitation confirmed a direct interaction between BATF2 and PTEN (Figure 6E), with their colocalization evident in AGS cells via immunofluorescence staining (Figure 6F). Further investigation into BATF2's impact on PTEN stability, post-cycloheximide treatment, showed that BATF2 overexpression notably extended PTEN protein's half-life (Figure 6G, Figure S7K). Meanwhile, our results indicated that inhibition of AKT/β-catenin signaling induced by BATF2 overexpression was partially abrogated by PTEN knockdown (Figure 6H). Furthermore, BATF2 silencing-induced activation of AKT/β-catenin axis was abolished by PTEN overexpression (Figure 6I). Consistently, the increasing sensitivity of GC cells to 5-Fu induced by BATF2 overexpression was attenuated by PTEN knockdown, whereas the resistance of GC cells to 5-Fu by BATF2 silencing was hampered by PTEN overexpression (Figure S7L-M). To investigate the specific binding sites of BATF2 and PTEN, we created three distinct truncation mutants of PTEN sequence, including mutant #1 (14-185aa), mutant #2 (190-350aa), and mutant #3 (352-403aa). Co-immunoprecipitation showed that BATF2 was detected in the eluted samples harboring a variant (14-185 amino acids) of PTEN, suggesting that PTEN binds to BATF2 through the domain (14-185aa) (Figure 6J). We further examined how BATF2 affected PTEN protein stability. The raise in PTEN degradation was reversed by the proteasome inhibitor MG-132 (Figure 6K-L). BATF2 knockdown increased PTEN ubiquitination, whereas BATF2 overexpression decreased it compared with the controls (Figure 6M-N). Therefore, BATF2 inhibits PTEN degradation through the ubiquitin-proteasome system. These results highlight BATF2 blocks the expression of the ABCG2 protein through the PTEN/AKT/β-catenin pathway, thereby increasing chemotherapy sensitivity.

## Discussion

Cancer stem cells (CSCs) represent a unique subset of tumor cells, notable for their exceptional ability to drive tumorigenesis and self-renew 6. These cells adeptly adjust to changes in their environment and exhibit a heightened resistance to conventional chemotherapy compared to other tumor cells 19. Research has shown that a minor fraction of CSCs can initiate and sustain long-term tumor growth, contribute to tumor relapse, and exhibit resistance to chemotherapy 20, 21. After administering chemotherapy to any tumor type, a residual CSC-enriched population remains, which, despite its small size within the tumor, significantly impacts treatment outcomes and patient prognoses. Understanding the biology and underlying molecular mechanisms of gastric CSCs is crucial for developing strategies to enhance patient outcomes in gastric cancer. Previous investigations have highlighted the importance of certain markers, such as CD44, NANOG, and SOX2 20, 21, particularly in gastrointestinal cancers, for their roles in maintaining and regulating the CSC population.

Through employing western blotting, RT-PCR, and 3D spheroid formation assays, we identified a notable reduction in the levels of stem cell markers within gastric cancer spheroids when juxtaposed with adherent cell cultures. Concurrently, we observed an increase in BATF2 expression in these cells compared to controls, establishing a negative relationship between BATF2 levels and the expression of stem cell markers. Specifically, gastric cancer cells with enhanced BATF2 expression exhibited significantly lower levels of the cancer stem cell markers CD44, NANOG, and SOX2 than the control cells. Moreover, the capability of these BATF2-overexpressing cells to form spheroids was impaired. These results imply that modulating BATF2 expression could be a viable strategy to influence the properties of gastric cancer stem cells.

In our study, we observed that patients with higher BATF2 expression in an internal cohort demonstrated improved prognoses after postoperative chemotherapy, while those with lower levels of BATF2 showed poorer outcomes, irrespective of receiving treatment. This aligns with the recognized phenomenon where cancer stem cells exhibit notable chemotherapy resistance, a trait that can be inherent or induced by chemotherapy and radiotherapy. Post-treatment, tumors often harbor a residual cell population rich in cancer stem cells, which are likely to survive. In our cellular studies, gastric cancer cells with increased BATF2 expression had a lower IC_50_ value for 5-Fu, suggesting a heightened sensitivity to the chemotherapy compared to control cells. Additionally, RNA sequencing analysis indicated a substantial decrease in ABCG2 protein levels in BATF2-overexpressed cells relative to controls. ABCG2, an ATP-binding cassette (ABC) transporter, acts as an ATP-dependent pump with ATP-binding and transmembrane domains 18, 22, typical of the ABC superfamily, facilitating the extrusion of drugs from cells using ATP hydrolysis 23. Our data suggests that by downregulating ABCG2, BATF2 can potentially amplify the chemotherapeutic responsiveness of gastric cancer cells, offering a promising avenue for enhancing treatment efficacy.

Studies have established a significant relationship between the AKT/β-catenin signaling pathway and tumor drug resistance, distinguishing between primary drug resistance (PDR) and multidrug resistance (MDR) as two forms of resistance. PDR describes a tumor's resistance to initial treatment drugs, while MDR refers to a tumor's acquired resistance to a range of anticancer drugs with varying structures and mechanisms after initial resistance to a single chemotherapy agent. The AKT/β-catenin signaling pathway is notably connected to MDR development in various solid tumors, such as gastric cancer 24, 25, liver cancer 26, colon cancer 27, 28, breast cancer 29, and lung cancer. Targeting and inhibiting the AKT/β-catenin pathway can potentially reverse drug resistance in tumor cells. Our study demonstrates that the overactivation of the AKT/β-catenin pathway contributes to maintaining MDR in tumor cells, mediated by the ATP binding cassette family's transport functions and anti-apoptotic mechanisms.

GESA enrichment analysis of RNA sequencing data indicated a significant association between BATF2 and the AKT signaling pathway. Previous studies have demonstrated that BATF2 inhibited the phosphorylation of AKT by PI3K/AKT pathway, then suppressed cell migration capacity and enhanced the immune response to tumor cells 30, 31, suggesting that BATF2 played a significant regulatory role in the AKT signaling. Consistently, our further investigations revealed that BATF2 overexpression led to reduced phosphorylation of AKT, while BATF2 knockout resulted in increased phosphorylation, an effect that could be reversed by the AKT inhibitor MK2206. PTEN, a critical phosphatase with both protein and lipid phosphatase activities, acts as a key regulator in the AKT/β-catenin signaling pathway 32-34 and can be inactivated through ubiquitination. A strong link between BATF2 and PTEN was observed in the TCGA database. Immunofluorescence colocalization and co-immunoprecipitation assays verified the interaction between BATF2 and PTEN. From these findings, we hypothesize that BATF2's direct binding to PTEN prevents PTEN's ubiquitination and degradation, thereby inhibiting the AKT/β-catenin/ABCG2 signaling pathway. This inhibition is pivotal in reducing stem cell-like traits and overcoming chemotherapy resistance in gastric cancer cells.

## Supplementary Material

Supplementary figures and tables.

## References

[1] Bray F, Laversanne M, Sung H, Ferlay J, Siegel RL, Soerjomataram I (2024). Global cancer statistics 2022: GLOBOCAN estimates of incidence and mortality worldwide for 36 cancers in 185 countries. CA Cancer J Clin.

[2] Fatehullah A, Terakado Y, Sagiraju S, Tan TL, Sheng T, Tan SH (2021). A tumour-resident Lgr5(+) stem-cell-like pool drives the establishment and progression of advanced gastric cancers. Nat Cell Biol.

[3] Hayakawa Y, Nakagawa H, Rustgi AK, Que J, Wang TC (2021). Stem cells and origins of cancer in the upper gastrointestinal tract. Cell Stem Cell.

[4] Dean M, Fojo T, Bates S (2005). Tumour stem cells and drug resistance. Nature reviews Cancer.

[5] Lytle N, Barber A, Reya T (2018). Stem cell fate in cancer growth, progression and therapy resistance. Nature reviews Cancer.

[6] Saygin C, Matei D, Majeti R, Reizes O, Lathia JD (2019). Targeting Cancer Stemness in the Clinic: From Hype to Hope. Cell Stem Cell.

[7] Meacham CE, Morrison SJ (2013). Tumour heterogeneity and cancer cell plasticity. Nature.

[8] Dorsey MJ, Tae HJ, Sollenberger KG, Mascarenhas NT, Johansen LM, Taparowsky EJ (1995). B-ATF: a novel human bZIP protein that associates with members of the AP-1 transcription factor family. Oncogene.

[9] Hasegawa H, Utsunomiya Y, Kishimoto K, Tange Y, Yasukawa M, Fujita S (1996). SFA-2, a novel bZIP transcription factor induced by human T-cell leukemia virus type I, is highly expressed in mature lymphocytes. Biochem Biophys Res Commun.

[10] Su ZZ, Lee SG, Emdad L, Lebdeva IV, Gupta P, Valerie K (2008). Cloning and characterization of SARI (suppressor of AP-1, regulated by IFN). Proc Natl Acad Sci U S A.

[11] Kayama H, Tani H, Kitada S, Opasawatchai A, Okumura R, Motooka D (2019). BATF2 prevents T-cell-mediated intestinal inflammation through regulation of the IL-23/IL-17 pathway. Int Immunol.

[12] Roy S, Guler R, Parihar SP, Schmeier S, Kaczkowski B, Nishimura H (2015). Batf2/Irf1 induces inflammatory responses in classically activated macrophages, lipopolysaccharides, and mycobacterial infection. J Immunol.

[13] Dash R, Su ZZ, Lee SG, Azab B, Boukerche H, Sarkar D (2010). Inhibition of AP-1 by SARI negatively regulates transformation progression mediated by CCN1. Oncogene.

[14] Dai L, Cui X, Zhang X, Cheng L, Liu Y, Yang Y (2016). SARI inhibits angiogenesis and tumour growth of human colon cancer through directly targeting ceruloplasmin. Nat Commun.

[15] Xie JW, Huang XB, Chen QY, Ma YB, Zhao YJ, Liu LC (2020). m(6)A modification-mediated BATF2 acts as a tumor suppressor in gastric cancer through inhibition of ERK signaling. Mol Cancer.

[16] Lin M, Tu RH, Wu SZ, Zhong Q, Weng K, Wu YK (2024). Increased ONECUT2 induced by Helicobacter pylori promotes gastric cancer cell stemness via an AKT-related pathway. Cell Death Dis.

[17] Zhong Q, Wang HG, Yang JH, Tu RH, Li AY, Zeng GR (2023). Loss of ATOH1 in Pit Cell Drives Stemness and Progression of Gastric Adenocarcinoma by Activating AKT/mTOR Signaling through GAS1. Adv Sci (Weinh).

[18] Yu Q, Ni D, Kowal J, Manolaridis I, Jackson SM, Stahlberg H (2021). Structures of ABCG2 under turnover conditions reveal a key step in the drug transport mechanism. Nat Commun.

[19] Wang Q, Liang N, Yang T, Li Y, Li J, Huang Q (2021). DNMT1-mediated methylation of BEX1 regulates stemness and tumorigenicity in liver cancer. J Hepatol.

[20] Tsao AN, Chuang YS, Lin YC, Su Y, Chao TC (2022). Dinaciclib inhibits the stemness of two subtypes of human breast cancer cells by targeting the FoxM1 and Hedgehog signaling pathway. Oncol Rep.

[21] Zhan Y, Chen Z, He S, Gong Y, He A, Li Y (2020). Long non-coding RNA SOX2OT promotes the stemness phenotype of bladder cancer cells by modulating SOX2. Mol Cancer.

[22] Taylor NMI, Manolaridis I, Jackson SM, Kowal J, Stahlberg H, Locher KP (2017). Structure of the human multidrug transporter ABCG2. Nature.

[23] Orlando BJ, Liao M (2020). ABCG2 transports anticancer drugs via a closed-to-open switch. Nat Commun.

[24] Jiang L, Chen Y, Min G, Wang J, Chen W, Wang H (2021). Bcl2-associated athanogene 4 promotes the invasion and metastasis of gastric cancer cells by activating the PI3K/AKT/NF-kappaB/ZEB1 axis. Cancer Lett.

[25] Wang C, Yang Z, Xu E, Shen X, Wang X, Li Z (2021). Apolipoprotein C-II induces EMT to promote gastric cancer peritoneal metastasis via PI3K/AKT/mTOR pathway. Clin Transl Med.

[26] Liu B, Fang X, Kwong DL, Zhang Y, Verhoeft K, Gong L (2022). Targeting TROY-mediated P85a/AKT/TBX3 signaling attenuates tumor stemness and elevates treatment response in hepatocellular carcinoma. J Exp Clin Cancer Res.

[27] Mangiapane LR, Nicotra A, Turdo A, Gaggianesi M, Bianca P, Di Franco S (2022). PI3K-driven HER2 expression is a potential therapeutic target in colorectal cancer stem cells. Gut.

[28] Narayanan S, Gujarati NA, Wang JQ, Wu ZX, Koya J, Cui Q (2021). The Novel Benzamide Derivative, VKNG-2, Restores the Efficacy of Chemotherapeutic Drugs in Colon Cancer Cell Lines by Inhibiting the ABCG2 Transporter. Int J Mol Sci.

[29] Wang L, Zhou Y, Jiang L, Lu L, Dai T, Li A (2021). CircWAC induces chemotherapeutic resistance in triple-negative breast cancer by targeting miR-142, upregulating WWP1 and activating the PI3K/AKT pathway. Mol Cancer.

[30] Liu J, Li J, Tuo Z, Hu W, Liu J (2023). BATF2 inhibits PD-L1 expression and regulates CD8+ T-cell infiltration in non-small cell lung cancer. J Biol Chem.

[31] Wang Q, Lu W, Yin T, Lu L (2019). Calycosin suppresses TGF-beta-induced epithelial-to-mesenchymal transition and migration by upregulating BATF2 to target PAI-1 via the Wnt and PI3K/Akt signaling pathways in colorectal cancer cells. J Exp Clin Cancer Res.

[32] Dai Y, Wan Y, Qiu M, Wang S, Pan C, Wang Y (2018). lncRNA MEG3 Suppresses the Tumorigenesis of Hemangioma by Sponging miR-494 and Regulating PTEN/ PI3K/AKT Pathway. Cell Physiol Biochem.

[33] Luongo F, Colonna F, Calapa F, Vitale S, Fiori ME, De Maria R (2019). PTEN Tumor-Suppressor: The Dam of Stemness in Cancer. Cancers (Basel).

[34] Shen WC, Lai YC, Li LH, Liao K, Lai HC, Kao SY (2019). Methylation and PTEN activation in dental pulp mesenchymal stem cells promotes osteogenesis and reduces oncogenesis. Nat Commun.

