# Supplementary material for: BATF2 inhibits the stem cell-like properties and chemoresistance of gastric cancer cells through PTEN/AKT/β-catenin pathway
Source: Theranostics. 2024 Oct 21;14(18):7007–22. doi: 10.7150/thno.98389 (PMC11610130; doi:10.7150/thno.98389)
Supplement: Supplementary file 1 — Supplementary figures and tables. [file thnov14p7007s1.pdf]

Supplementary Figure legends

Figure S1. scRNA-seq analysis of 9 GC samples. (A) A violin plot illustrating the expression levels of specific marker genes across nine distinct cell types. (B) Featureplot showing the distribution of clusters as indicated by specific marker genes. (C) GSEA was performed on scRNA-seq data comparing the BATF2 low expression cells to the high expression epithelial cells.

Figure S2. mRNA expression levels of stemness markers in adherent cells and spheroid. (A) Analysis of stem cell marker expression in adherent and spheroid cells via qRT-PCR. (B) Examination of stem cell markers in HGC-27 and AGS cells with BATF2 overexpression or knockdown using qRT-PCR. (C) qRT-PCR assessment of stem cell markers in HGC-27 and AGS spheroid with elevated BATF2 expression.

Figure S3. BATF2 knockdown enhances stem cell-like properties in gastric cancer cells. (A) Western blot analysis evaluating stem cell marker levels in HGC-27 and AGS cells with stable BATF2 knockdown, with or without MK2206 treatment. (B) Western blot examination of stem cell markers and key AKT pathway components in spheroid from HGC-27 and AGS with stable BATF2 knockdown. (C) Images showcasing spheroids with or without BATF2 knockdown. (D-E) Immunofluorescence imaging and quantification of CD44 and NANOG in spheroids treated with BATF2 knockdown and MK2206. (F-G) Flow cytometric assessment of the CD44-positive cell fraction in HGC-27 or AGS cells with stable transfection, including visual representations and numerical quantification of the findings.

Figure S4. BATF2 impedes gastric cancer cell stem cell-like properties in MKN-45 cells.

(A) Analysis of BATF2 expression GES-1, MKN-45, HGC-27, ASG and MKN-28 cells via western blot. (B-C) Western blot assessment of stem cell markers and AKT pathway components in MKN-45 cells with elevated BATF2 expression. (D-F) Spheroid formation frequency in U-bottom 96-well plates using MKN-45 cells with varying BATF2 expression. (G-I) Serial dilution and subcutaneous xenograft of MKN-45 cells with/without BATF2 overexpression into NOD/SCID mice, tracking tumor cell injection numbers and tumor formation frequency by day 42, with probability estimates from Extreme Limiting Dilution Analysis (ELDA).

Figure S5. BATF2 increased the sensitivity of 5-Fu treatment.

(A) Kaplan–Meier survival analysis assessing chemotherapy's impact based on BATF2 high and low expression patients in external cohort. (B) Western blot assessment of stem cell markers and AKT pathway components in parental and 5-Fu resistant cells. (C) Concentration-survival curves for 5-Fu in 5-Fu resistant AGS and HGC-27 cell lines determined by CCK-8 assay. (D) The body weight of all mice involved in Figure 5H during the experiment.

Figure S6. Analysis of genes downstream of BATF2.

(A) Sequencing data indicating ABCG2 as a gene regulated by BATF2. (B) Expression levels of ABC transporter family members derived from sequencing. (C) Gene set enrichment analysis (GSEA) conducted to understand the downstream signaling impacted by BATF2.

Figure S7. BATF2 increases 5-Fu sensitivity via blocking PTEN/AKT/β-catenin/ABCG2 signaling.

(A) Western blot analysis of ABCG2, ABCB6, and ABCB1 in HGC-27 cells with increased BATF2 expression. (B) qRT-PCR evaluation of ABCG2, ABCB6, and ABCB1 in BATF2-altered AGS and HGC-27 cells. (C) Feature plot depiction of cells positive for ABCG2 (red), BATF2 (green), or both (yellow). (D) Western blot results for ABCG2 and BATF2 expression in GC cell lines administrated by gradient 5-Fu treatment. (E) Western blot analysis of AKT signaling components in BATF2-modified HGC-27 cells. (F) Protein level assessment of AKT pathway members in HGC-27 cells with BATF2 knockdown and MK2206 treatment. (G) Immunofluorescence staining of β-catenin and EpCAM in GC organoids, with BATF2 knockdown or overexpression (DAPI-stained nuclei). (H) Quantification of nuclear β-catenin fluorescence in G. (I) Nuclear and cytoplasmic protein extractions assay of phosphorylated β-catenin and total β-catenin induced by BATF2 overexpression. (J) qRT-PCR evaluation of BATF2 and PLHPP in BATF2-altered AGS and HGC-27 cells. (K) Quantification of PTEN expression in AGS cells after cycloheximide treatment. (L) Concentration-survival curves for 5-Fu in AGS and HGC-27 cell lines with BATF2 overexpression and PTEN deletion determined by CCK-8 assay. (M) Concentration-survival curves for 5-Fu in AGS and HGC-27 cell lines with BATF2 deletion and PTEN overexpression determined by CCK-8 assay.

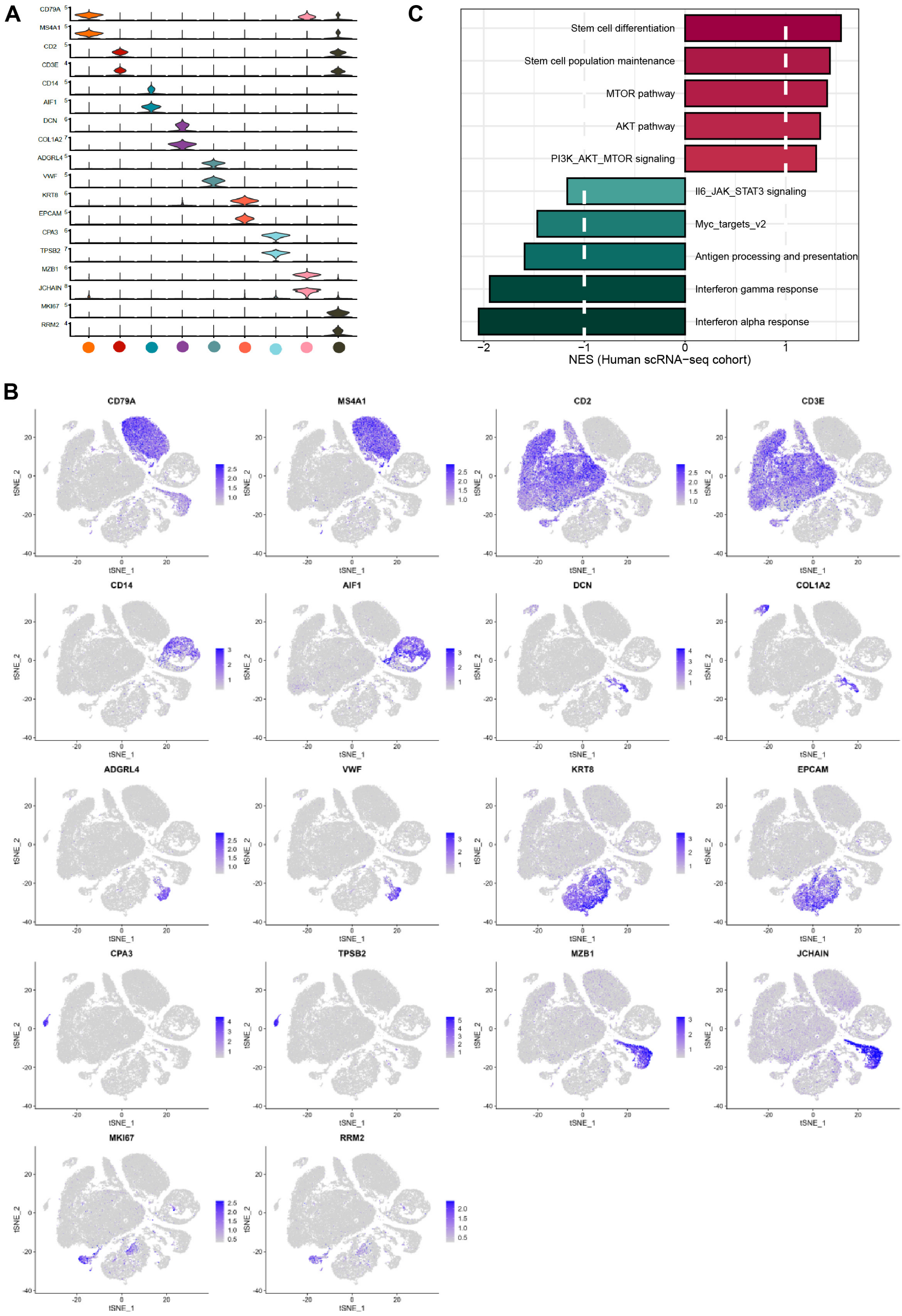
A
CD79A
MS4A1
CD2
CD3E
CD14
AIF1
DCN
COL1A2
ADGRL4
VWF
KRT8
EPCAM
CPA3
TPSB2
MZB1
JCHAIN
MKI67
RRM2
C
Stem cell differentiation
Stem cell population maintenance
MTOR pathway
AKT pathway
PI3K_AKT_MTOR signaling
Il6_JAK_STAT3 signaling
Myc_targets_v2
Antigen processing and presentation
Interferon gamma response
Interferon alpha response
NES (Human scRNA−seq cohort)
B
CD79A
MS4A1
CD2
CD3E
CD14
AIF1
DCN
COL1A2
ADGRL4
VWF
KRT8
EPCAM
CPA3
TPSB2
MZB1
JCHAIN
MKI67
RRM2
tSNE_1
tSNE_2

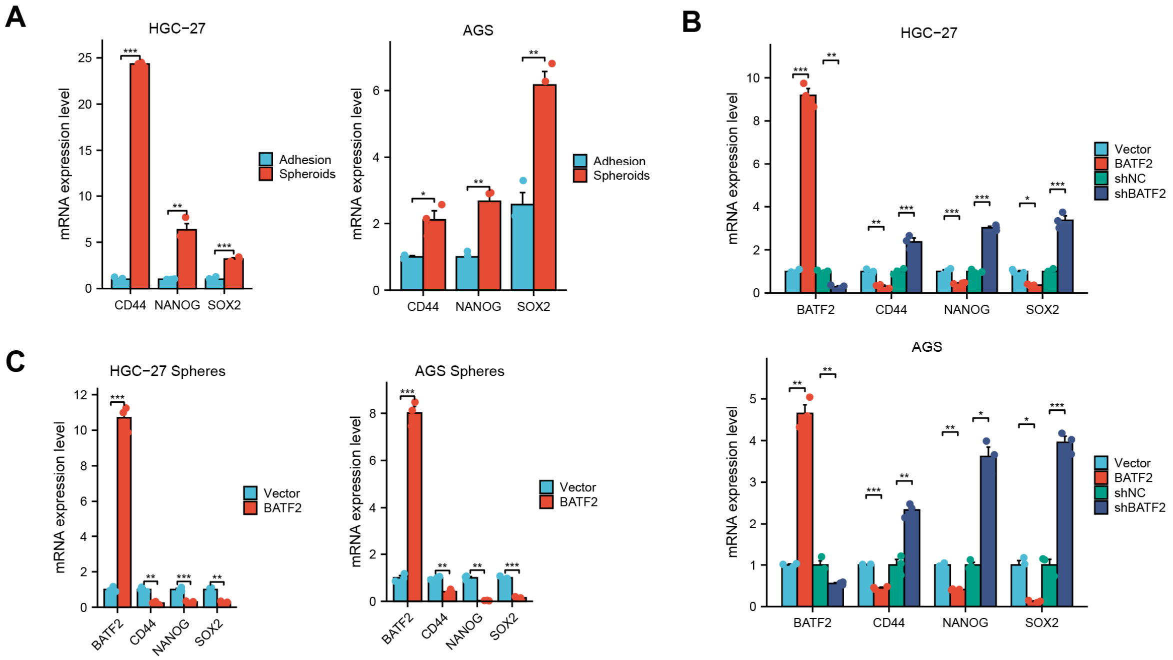
A
HGC−27
mRNA expression level
CD44
NANOG
SOX2
Adhesion
Spheroids
AGS
B
HGC−27
Vector
BATF2
shNC
shBATF2
AGS
C
HGC-27 Spheres
AGS Spheres

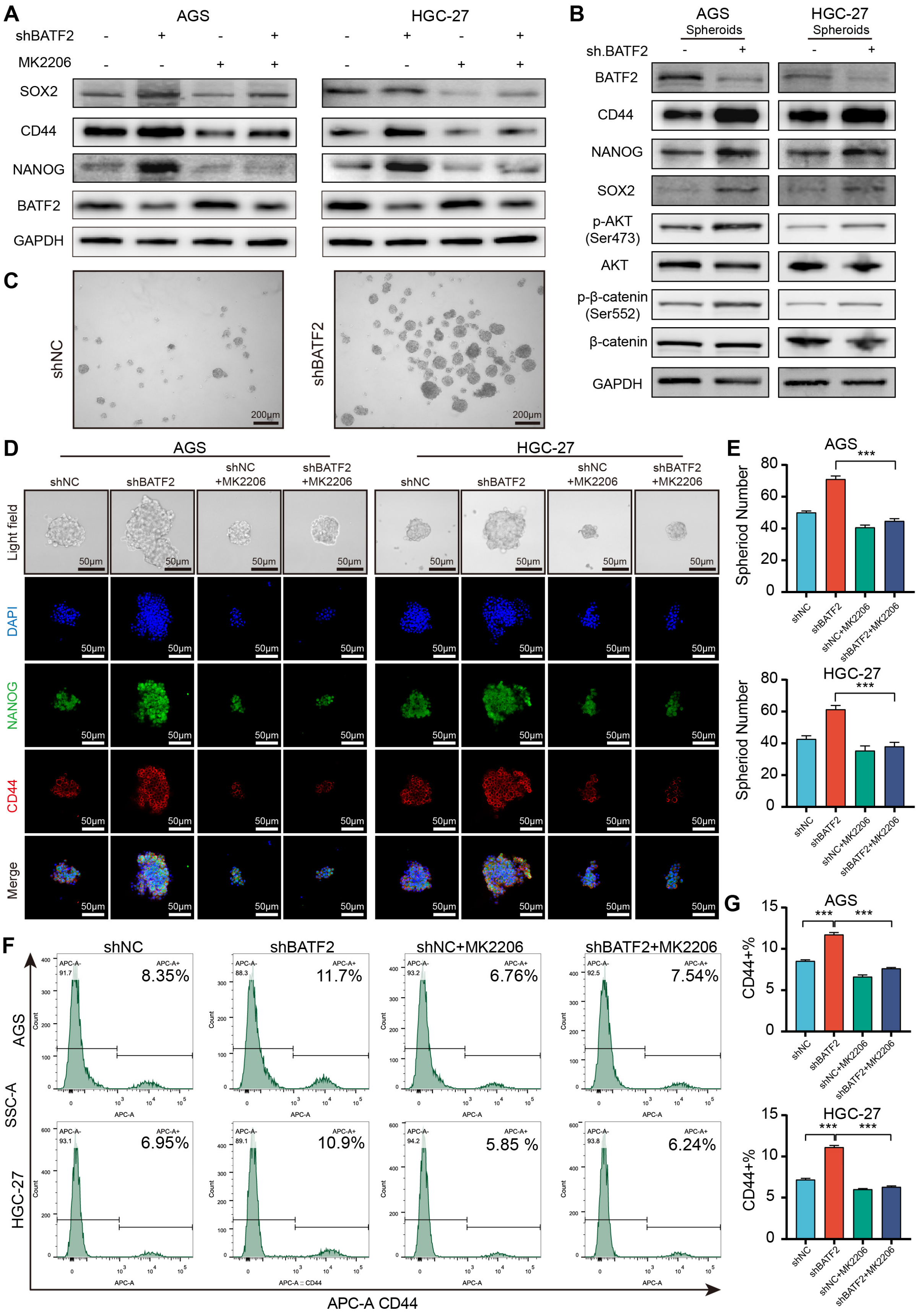
A
AGS
HGC-27
shBATF2
MK2206
SOX2
CD44
NANOG
BATF2
GAPDH
B
AGS Spheroids
HGC-27 Spheroids
sh.BATF2
BATF2
CD44
NANOG
SOX2
p-AKT (Ser473)
AKT
p-β-catenin (Ser552)
β-catenin
GAPDH
C
shNC
shBATF2
200μm
D
AGS
HGC-27
shNC
shBATF2
shNC +MK2206
shBATF2 +MK2206
Light field
DAPI
NANOG
CD44
Merge
50μm
E
AGS
HGC-27
Spheriod Number
shNC
shBATF2
shNC+MK2206
shBATF2+MK2206
***
F
shNC
shBATF2
shNC+MK2206
shBATF2+MK2206
AGS
HGC-27
SSC-A
APC-A CD44
8.35%
11.7%
6.76%
7.54%
6.95%
10.9%
5.85 %
6.24%
G
AGS
HGC-27
CD44+%
***

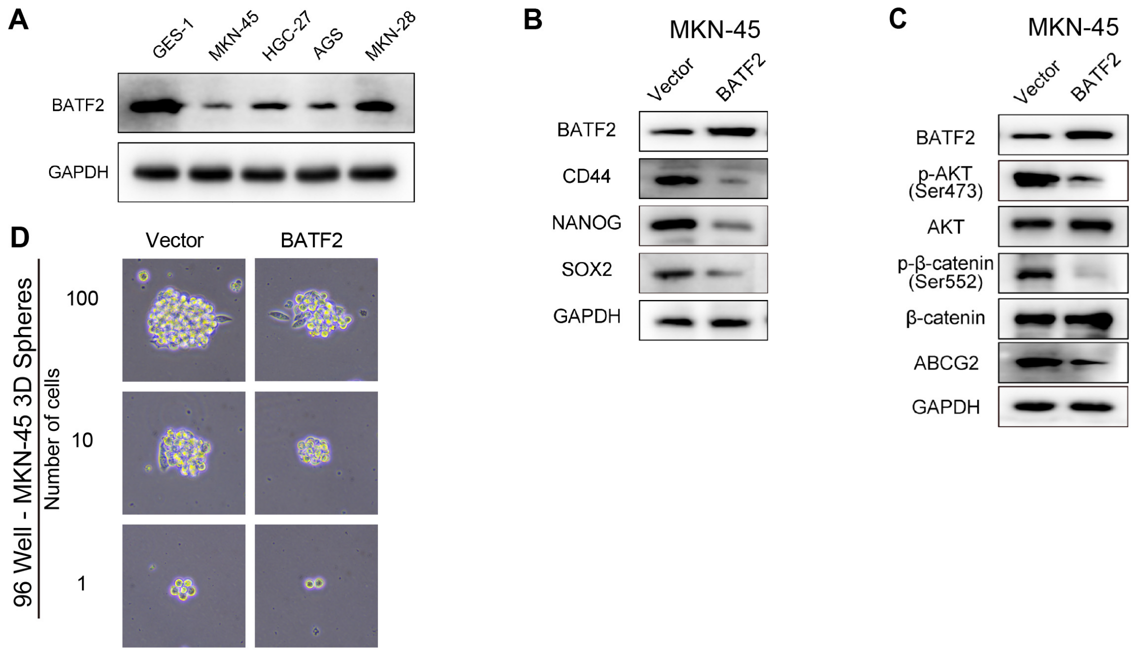

E

Cell Number	Vector		BATF2	
	Number of well	Days	Number of well	Days
100	32/32	10	32/32	10
10	32/32		29/32	
1	26/32		17/32	
P value	< .0001			

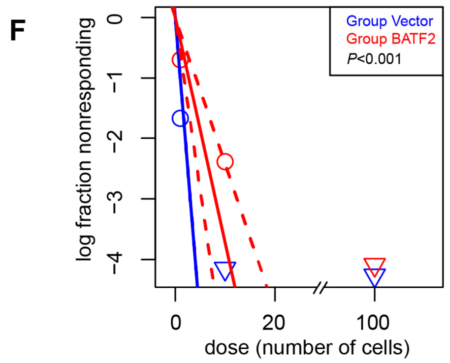

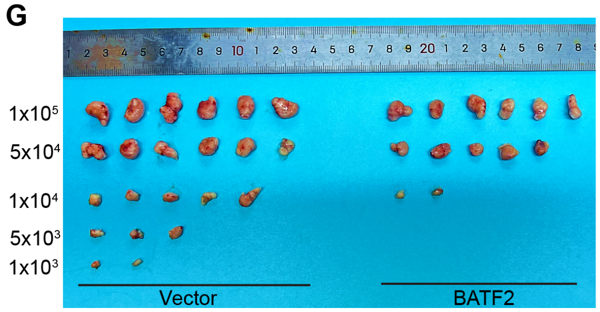

H

Cell Dose	Vector		BATF2	
	Tumors/N	Days	Tumors/N	Days
100000	6/6	42	6/6	42
50000	6/6		5/6	
10000	5/6		2/6	
5000	3/6		0/6	
1000	2/6		0/6	

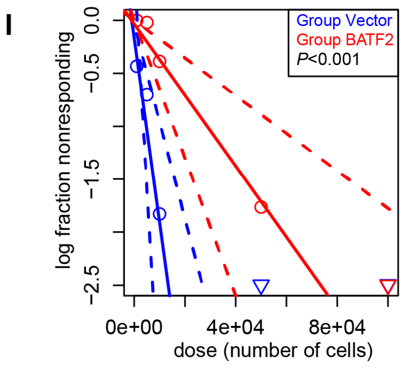

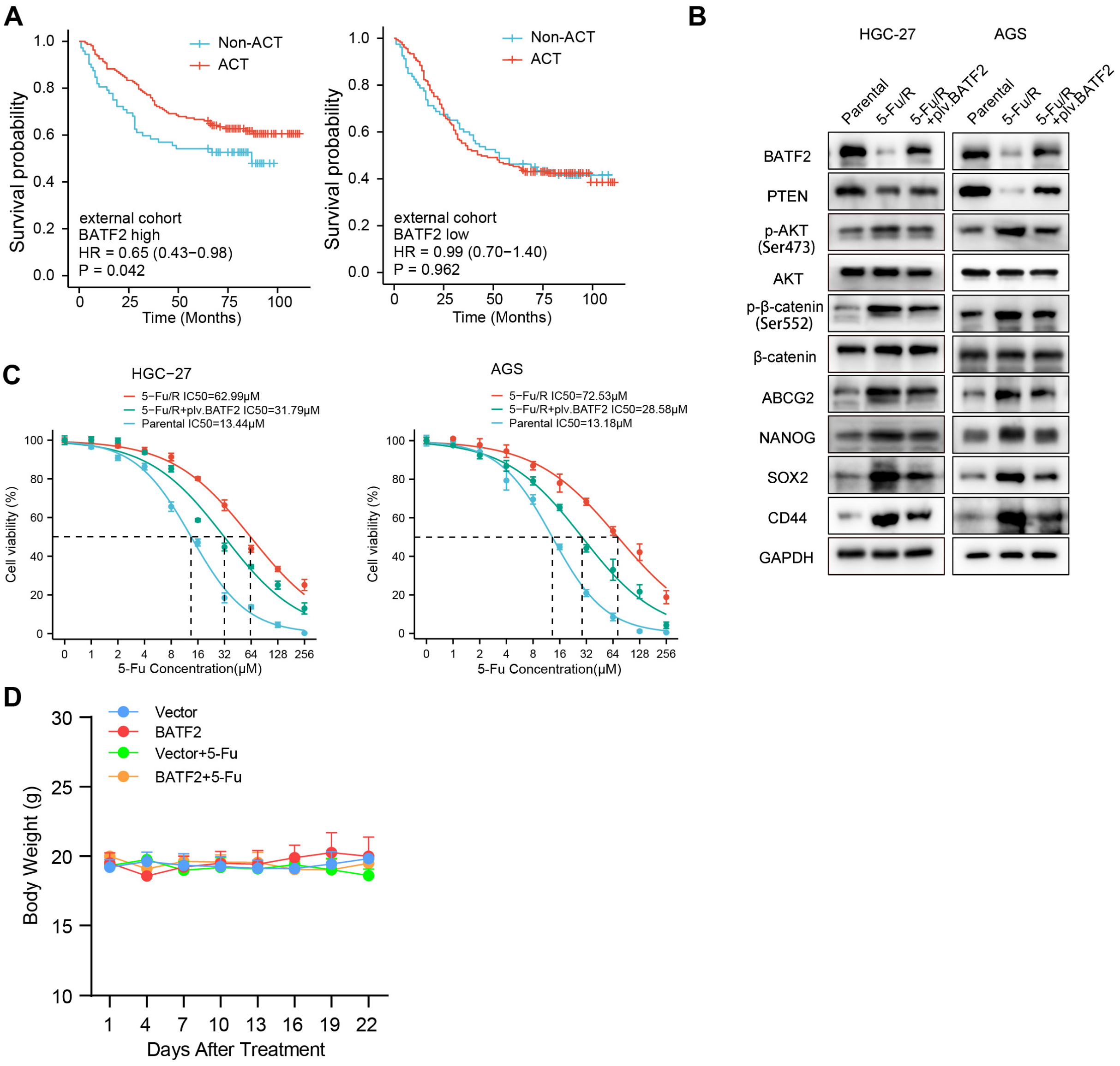
A
Non-ACT
ACT
Survival probability
external cohort
BATF2 high
HR = 0.65 (0.43−0.98)
P = 0.042
Time (Months)
external cohort
BATF2 low
HR = 0.99 (0.70−1.40)
P = 0.962
B
HGC-27
AGS
Parental
5-Fu/R
5-Fu/R +plv.BATF2
BATF2
PTEN
p-AKT (Ser473)
AKT
p-β-catenin (Ser552)
β-catenin
ABCG2
NANOG
SOX2
CD44
GAPDH
C
HGC−27
5−Fu/R IC50=62.99μM
5−Fu/R+plv.BATF2 IC50=31.79μM
Parental IC50=13.44μM
AGS
5−Fu/R IC50=72.53μM
5−Fu/R+plv.BATF2 IC50=28.58μM
Parental IC50=13.18μM
Cell viability (%)
5-Fu Concentration(μM)
D
Vector
BATF2
Vector+5-Fu
BATF2+5-Fu
Body Weight (g)
Days After Treatment

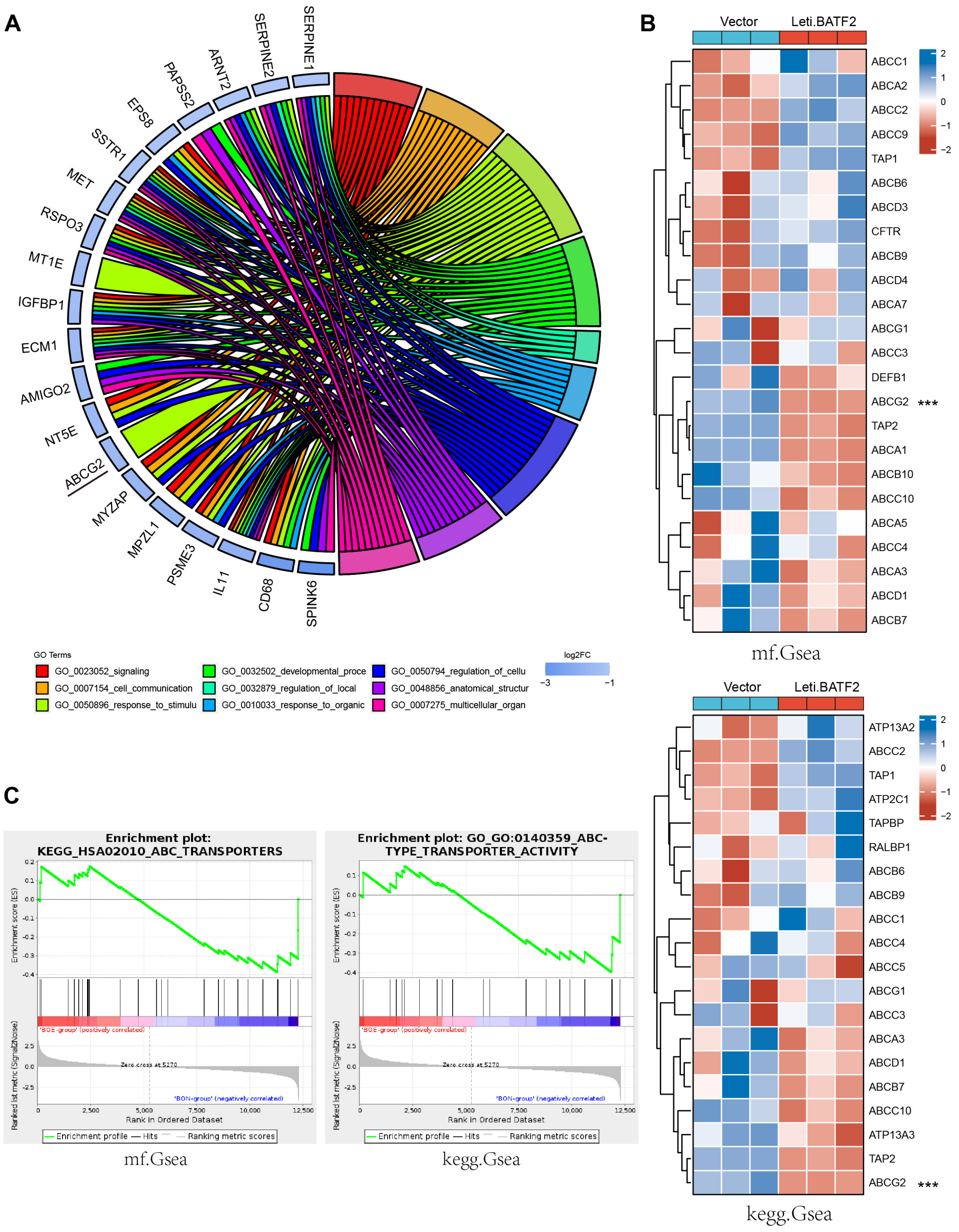
A
B
C
SERPINE1
SERPINE2
ARNT2
PAPSS2
EPS8
SSTR1
MET
RSPO3
MT1E
IGFBP1
ECM1
AMIGO2
NT5E
ABCG2
MYZAP
MPZL1
PSME3
IL11
CD68
SPINK6
GO Terms
GO_0023052_signaling
GO_0007154_cell_communication
GO_0050896_response_to_stimulu
GO_0032502_developmental_proce
GO_0032879_regulation_of_local
GO_0010033_response_to_organic
GO_0050794_regulation_of_cellu
GO_0048856_anatomical_structur
GO_0007275_multicellular_organ
log2FC
-3
-1
Vector
Leti.BATF2
ABCC1
ABCA2
ABCC2
ABCC9
TAP1
ABCB6
ABCD3
CFTR
ABCB9
ABCD4
ABCA7
ABCG1
ABCC3
DEFB1
ABCG2 ***
TAP2
ABCA1
ABCB10
ABCC10
ABCA5
ABCC4
ABCA3
ABCD1
ABCB7
2
1
0
−1
−2
mf.Gsea
Vector
Leti.BATF2
ATP13A2
ABCC2
TAP1
ATP2C1
TAPBP
RALBP1
ABCB6
ABCB9
ABCC1
ABCC4
ABCC5
ABCG1
ABCC3
ABCA3
ABCD1
ABCB7
ABCC10
ATP13A3
TAP2
ABCG2 ***
kegg.Gsea
Enrichment plot: KEGG_HSA02010_ABC_TRANSPORTERS
Enrichment score (ES)
'BOE-group' (positively correlated)
Zero cross at 5270
'BON-group' (negatively correlated)
Ranked list metric (Signal2Noise)
Rank in Ordered Dataset
Enrichment profile — Hits — Ranking metric scores
mf.Gsea
Enrichment plot: GO_GO:0140359_ABC-TYPE_TRANSPORTER_ACTIVITY
Enrichment score (ES)
'BOE-group' (positively correlated)
Zero cross at 5270
'BON-group' (negatively correlated)
Ranked list metric (Signal2Noise)
Rank in Ordered Dataset
Enrichment profile — Hits — Ranking metric scores
kegg.Gsea

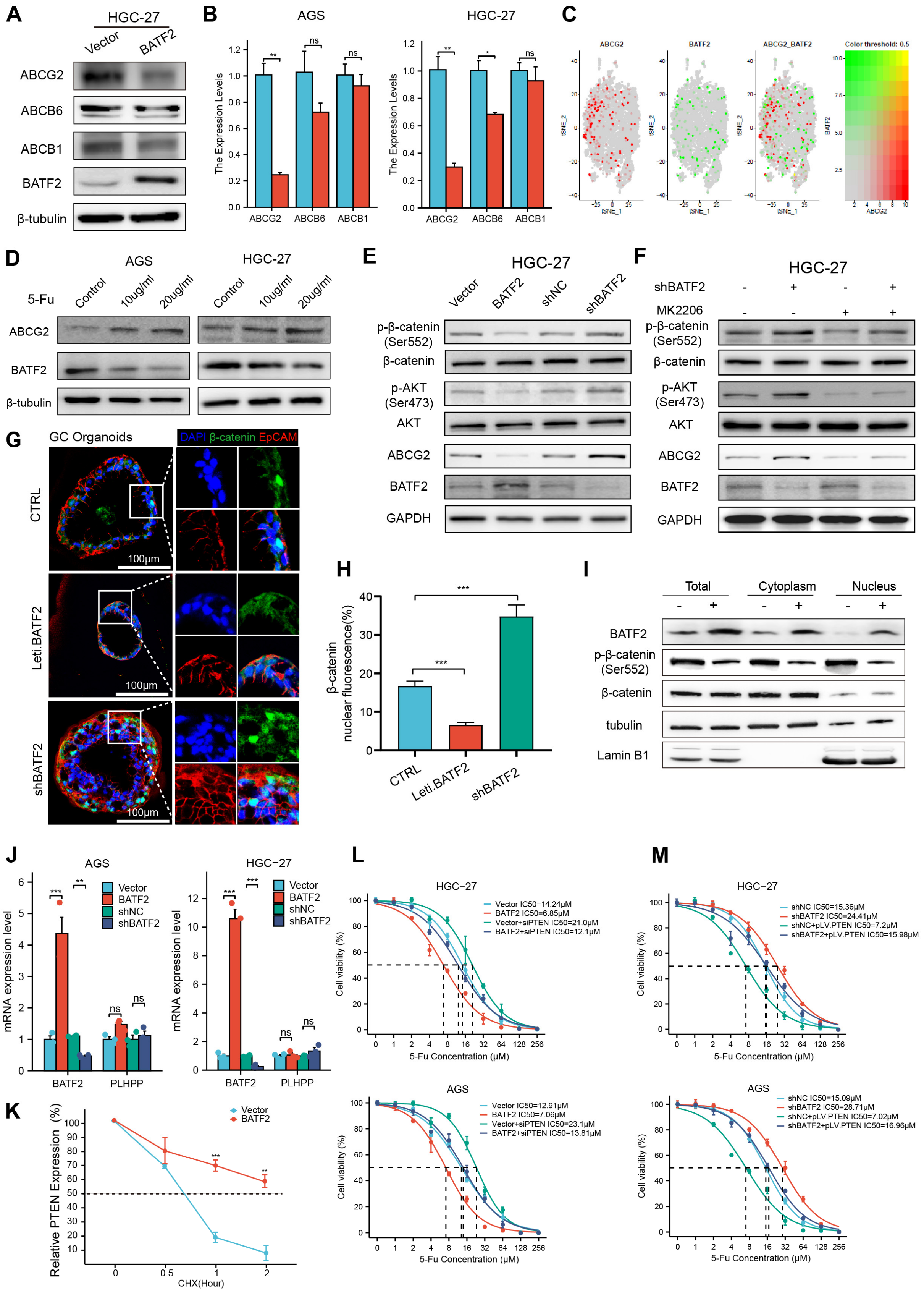

A
HGC-27
Vector
BATF2
ABCG2
ABCB6
ABCB1
BATF2
β-tubulin
B
AGS
HGC-27
The Expression Levels
ABCG2
ABCB6
ABCB1
**
ns
*
C
ABCG2
BATF2
ABCG2_BATF2
Color threshold: 0.5
tSNE_1
tSNE_2
D
AGS
HGC-27
5-Fu
Control
10ug/ml
20ug/ml
ABCG2
BATF2
β-tubulin
E
HGC-27
Vector
BATF2
shNC
shBATF2
p-β-catenin (Ser552)
β-catenin
p-AKT (Ser473)
AKT
ABCG2
BATF2
GAPDH
F
HGC-27
shBATF2
MK2206
p-β-catenin (Ser552)
β-catenin
p-AKT (Ser473)
AKT
ABCG2
BATF2
GAPDH
G
GC Organoids
DAPI β-catenin EpCAM
CTRL
Leti.BATF2
shBATF2
100μm
H
β-catenin nuclear fluorescence(%)
***
CTRL
Leti.BATF2
shBATF2
I
Total
Cytoplasm
Nucleus
BATF2
p-β-catenin (Ser552)
β-catenin
tubulin
Lamin B1
J
AGS
HGC-27
mRNA expression level
Vector
BATF2
shNC
shBATF2
BATF2
PLHPP
***
**
ns
K
Vector
BATF2
Relative PTEN Expression (%)
CHX(Hour)
***
**
L
HGC-27
Vector IC50=14.24μM
BATF2 IC50=6.85μM
Vector+siPTEN IC50=21.0μM
BATF2+siPTEN IC50=12.1μM
AGS
Vector IC50=12.91μM
BATF2 IC50=7.06μM
Vector+siPTEN IC50=23.1μM
BATF2+siPTEN IC50=13.81μM
Cell viability (%)
5-Fu Concentration (μM)
M
HGC-27
shNC IC50=15.36μM
shBATF2 IC50=24.41μM
shNC+pLV.PTEN IC50=7.2μM
shBATF2+pLV.PTEN IC50=15.98μM
AGS
shNC IC50=15.09μM
shBATF2 IC50=28.71μM
shNC+pLV.PTEN IC50=7.02μM
shBATF2+pLV.PTEN IC50=16.96μM
Cell viability (%)
5-Fu Concentration (μM)

Table S1. List of Antibodies

REAGENT or RESOURCE	SOURCE	IDENTIFIER
Antibodies		
Phospho-Akt (Ser473) (D9E) XP® Rabbit mAb	CST	Cat. # 4060s
Akt Antibody #9272 Rabbit mAb	CST	Cat. # 9272S
Phospho-β-Catenin (Ser552) (D8E11) Rabbit mAb	CST	Cat. # 5651
β-Catenin (D10A8) XP® Rabbit mAb	CST	Cat. # 8480s
ABCG2 (D5V2K) XP® Rabbit mAb	CST	Cat. # 42078s
GAPDH (14C10) Rabbit mAb #2118	CST	Cat. # 2118s
BATF2 Antibody (1B11)	Santa cruz	Cat. # sc-293274
PTEN (138G6) Rabbit mAb	CST	Cat. # 9559S
β3-Tubulin (D71G9) XP® Rabbit mAb	CST	Cat. # 5568S
Anti-Laminin beta 1 antibody	abcam	Cat. # ab69633
Sox2 (D9B8N) Rabbit mAb	CST	Cat. # 23064S
Sox9 (D8G8H) Rabbit mAb	CST	Cat. # 82630
Recombinant Human CD44 Protein	abcam	Cat. # RP00973
Anti-PHLPP1 antibody[EPR27151-55]	abcam	Cat. # ab305295
Anti-Nanog antibody [EPR2027(2)]	abcam	Cat. # ab109250
Anti-ABCB6/PRP antibody [EPR21891]	abcam	Cat. # ab221159
MDR1/ABCB1 (E1Y7S) Rabbit mAb	CST	Cat. # 13978s
CD326 (EpCAM) Monoclonal Antibody (1B7)	Thermofisher	Cat. # 14-9326-82
Goat anti-Rabbit IgG (H+L) Cross-Adsorbed Secondary Antibody, Alexa Fluor™ 488	Thermofisher	Cat. # A-11008
Goat anti-Mouse IgG (H+L) Cross-Adsorbed Secondary Antibody, Alexa Fluor™ 488	Thermofisher	Cat. # A-11001
Goat anti-Rabbit IgG (H+L) Cross-Adsorbed Secondary Antibody, Alexa Fluor™ 568	Thermofisher	Cat. # A-11011
Goat anti-Mouse IgG (H+L) Cross-Adsorbed Secondary Antibody, Alexa Fluor™ 568	Thermofisher	Cat. # A-11004

Table S2. Clinical characteristics of each sample for scRNA-seq

Sample	Age	Geder	Histopathological diagnosis	Tumor site	Lauren's classification	Pathological stage
Sample01	55	F	Moderately poorly differentiated adenocarcinoma	Antrum	Mixed	pT2N3aM0
Sample02	70	M	Poorly differentiated adenocarcinoma, partial signet ring cell carcinoma	Cardia	Mixed	pT3N3aM0
Sample03	63	M	Signet ring cell carcinoma	Antrum	Diffuse	pT4aN2M0
Sample04	59	M	Poorly differentiated adenocarcinoma	Corpus	Diffuse	pT2N0M0
Sample05	65	M	Moderately differentiated adenocarcinoma	Corpus	Intestinal	pT2N0M0
Sample06	62	F	Poorly differentiated adenocarcinoma	Corpus	Diffuse	pT4aN1M0
Sample07	69	M	Moderately differentiated adenocarcinoma	Corpus	Intestinal	pT2N1M0
Sample08	62	M	Moderately poorly differentiated adenocarcinoma	Cardia	Mixed	pT3N2M0
Sample09	53	F	Moderately differentiated adenocarcinoma	Antrum	Intestinal	pT2N1M0
